# Rhythmic Relating: Bidirectional Support for Social Timing in Autism Therapies

**DOI:** 10.3389/fpsyg.2022.793258

**Published:** 2022-05-16

**Authors:** Stuart Daniel, Dawn Wimpory, Jonathan T. Delafield-Butt, Stephen Malloch, Ulla Holck, Monika Geretsegger, Suzi Tortora, Nigel Osborne, Benjaman Schögler, Sabine Koch, Judit Elias-Masiques, Marie-Claire Howorth, Penelope Dunbar, Karrie Swan, Magali J. Rochat, Robin Schlochtermeier, Katharine Forster, Pat Amos

**Affiliations:** ^1^British Association of Play Therapists, London, United Kingdom; ^2^BCU Health Board (NHS), Bangor, United Kingdom; ^3^School of Human and Behavioural Sciences, Bangor University, Bangor, United Kingdom; ^4^Laboratory for Innovation in Autism, University of Strathclyde, Glasgow, United Kingdom; ^5^School of Education, University of Strathclyde, Glasgow, United Kingdom; ^6^Westmead Psychotherapy Program, School of Medicine, University of Sydney, Sydney, NSW, Australia; ^7^MARCS Institute for Brain, Behaviour and Development, Western Sydney University, Sydney, NSW, Australia; ^8^Music Therapy, Department of Communication and Psychology, Aalborg University, Aalborg, Denmark; ^9^The Grieg Academy Music Therapy Research Centre, NORCE Norwegian Research Centre, Bergen, Norway; ^10^Dancing Dialogue, LCAT, New York, NY, United States; ^11^Department of Music, University of Edinburgh, Edinburgh, United Kingdom; ^12^Perception Movement Action Research Consortium, University of Edinburgh, Edinburgh, United Kingdom; ^13^Research Institute for Creative Arts Therapies, Alanus University, Alfter, Germany; ^14^School of Therapy Sciences, Creative Arts Therapies, SRH University Heidelberg, Heidelberg, Germany; ^15^Independent Researcher, Glasgow, United Kingdom; ^16^Department of Counseling, Leadership, and Special Education, Missouri State University, Springfield, MO, United States; ^17^Functional and Molecular Neuroimaging Unit, IRCCS Istituto delle Scienze Neurologiche di Bologna, Bologna, Italy; ^18^Independent Researcher, London, United Kingdom; ^19^Independent Researcher, Ardmore, PA, United States

**Keywords:** autism, Rhythmic Relating, synchrony, sensorimotor integration, therapy, social timing, movement, Communicative Musicality

## Abstract

We propose *Rhythmic Relating* for autism: a system of supports for friends, therapists, parents, and educators; a system which aims to augment bidirectional communication and complement existing therapeutic approaches. We begin by summarizing the developmental significance of *social timing* and the *social-motor-synchrony* challenges observed in early autism. Meta-analyses conclude the early primacy of such challenges, yet cite the lack of focused therapies. We identify core relational parameters in support of social-motor-synchrony and systematize these using the *communicative musicality* constructs: *pulse*; *quality*; and *narrative*. *Rhythmic Relating* aims to augment the clarity, contiguity, and pulse-beat of spontaneous behavior by recruiting rhythmic supports (cues, accents, turbulence) and relatable vitality; facilitating the predictive flow and just-ahead-in-time planning needed for good-enough social timing. From here, we describe possibilities for playful therapeutic interaction, small-step co-regulation, and layered sensorimotor integration. Lastly, we include several clinical case examples demonstrating the use of *Rhythmic Relating* within four different therapeutic approaches (Dance Movement Therapy, Improvisational Music Therapy, Play Therapy, and Musical Interaction Therapy). These clinical case examples are introduced here and several more are included in the Supplementary Material (Examples of Rhythmic Relating in Practice). A suite of pilot intervention studies is proposed to assess the efficacy of combining *Rhythmic Relating* with different therapeutic approaches in playful work with individuals with autism. Further experimental hypotheses are outlined, designed to clarify the significance of certain key features of the *Rhythmic Relating* approach.

## Introduction

### Co-regulation: A Paradox at the Heart of Autism^1^

Some people with autism experience their authentic self as non-social. Here at the start, we wish to draw attention to the rights of the individual with autism to remain incommunicado and socially withdrawn. Can we, as people without autism, be okay with this? Some people with autism live with a longing to interact, so often denied or limited by the anxiety of sensory overwhelm and fracture.^1^ Can we be available but truly not demanding? With these terms comprising our ethical baseline, we aim to develop a system of relational skills for neurotypical communicators based on the needs, concerns, and specific sociabilities of people with autism.

For many people with autism the social world can be painful, fractured, overwhelming, and compulsive. Paradoxically though, the need to learn how to self-regulate and self-integrate often necessitates some level of *co-regulation* – a social phenomenon^2^.

From birth, typically-developing (TD) infants actively seek signals and experiences of safety from others (Porges, 2018); they are seeking regulation through shared experience (co-regulation), where *regulation* can be defined as: the ability to attain and maintain a *good-enough* (appropriate) state of arousal^3^ fit for task/environment/moment. Without safety and co-regulation, all infants become dysregulated. This can appear in behavior as over/under vigilance and response, fight/flight, agitation, shut down, avoidance, or seeking sensory stimulation/information.

Co-regulation is only possible when people feel safe together. Safety sets off a train of events. Initially, the nervous system either down-regulates the Mobilization response (fight/flight) or re-routes the Immobilization response (shut down/freeze) (ibid.). In either case, the next step activates the Social Engagement System, supporting ease and sociability (Porges et al., 1996; Porges, 2018). This is the basis of co-regulation: initial safety = social engagement increases = more safety = more engagement = …

Along with physical touch within the earliest attachment relationship, TD infant-parent *play* is a fundamental domain of co-regulation. During play, TD infants push the boundaries of safety, expanding the window through forays in and out of vulnerability – think of the uncertainty inherent in the games “hide-n-seek” and “peek-a-boo.” For co-regulation, what is important is the sense of traveling safely together through a familiar-enough narrative flow of play, from calm through to vulnerable and back again (Schore, 1994; Porges et al., 1996; Porges, 2021; Porges and Daniel, 2021). This co-regulatory play process is seen as an exercise for self-regulation and self-integration (Porges, 2021).

That sense of co-regulatory traveling together necessitates good-enough social timing. Yet, to differing degrees, individuals with autism often find themselves out of sync with other people. Children with autism have difficulty picking up subtleties of gesture from TD children (Rochat et al., 2013; Di Cesare et al., 2017a). TD adults have difficulty picking up subtleties of gesture from children with autism (Casartelli et al., 2020b). This bidirectional range of perceptual and motoric dissimilarity can lead to mutual misunderstanding. Asymmetries and asynchronies in the meeting of two people can lead to a mismatch in time-frame; the result: it is difficult to play together.

If we can tailor our communication toward social timing and safety, we may facilitate a gentle vagal feedback loop within the person with autism. Initial simplicity, sameness, and tailored communication may support a sense of safety, just a little… which will reduce anxiety and increase interactivity, just a little… which may lead to a short moment of interactive flow… which will reduce anxiety and increase interactivity, just a little…

Yet some children or individuals with autism may not be *ready* to interact. People with autism often have baseline sensory integration (SI) challenges (Ben-Sasson et al., 2019), including overwhelm or under-discrimination in tactile, visual, auditory, proprioceptive, and/or interoceptive fields. These challenges are neurosequentially primary to social engagement, leading to a primary feeling of wrongness, unsafety, and dysregulation. Here, any attempts at interaction may result in further withdrawal – a response to sensorimotor demand, SI challenge, or emotional overwhelm. Clearly, any support for playful co-regulation needs to address challenges of SI (Ben-Sasson et al., 2019), *as well as* those of Social Timing (Wimpory et al., 2002), and will often start by addressing the need for acclimatization, aloneness, simplicity, sameness, calm, short duration interaction, and rest. We suggest a fundamentally client-led basis to interactive play. This counteracts the potential ontological risk of coercing any child or individual with autism into neurotypical expectations for patterns of social engagement.

### Rhythmic Relating for Autism

In this paper we propose *Rhythmic Relating*, a system which aims to augment bidirectional communication and facilitate good-enough social timing; opening up the possibility of playful therapeutic interaction, small-step co-regulation, and layered sensorimotor integration. Our intention, in researching the model, was to take a fresh look at the current neuroscience and experimental psychology of rhythm, musical experience, movement, interaction, and autism. We wanted first, to be surprised by new parameters and then systematize these parameters as a working therapeutic tool-kit using solid concepts and practitioner experience from best-practice therapy (Dance Movement Therapy, Improvisational Music Therapy, Play Therapy, and Musical Interaction Therapy) and the *Communicative Musicality* model.

Rhythmic Relating focuses on interaction between neurotypical people and clients with *Autism with Therapeutic Needs* – Autism^(Therapeutic Needs)^. By this, we mean individuals with a classical autism phenotype^4^, and with *therapeutic needs which cause distress*^5^ (mental health, SI, and/or dysregulation); perhaps with co-morbid learning disability (LD); perhaps non-speakers or unconventional communicators.

Rhythmic Relating is not a stand-alone model. It can complement any therapeutic approach open to elements of person-led practice. We assume a sensitive, playful practitioner^6^ working with a single client^7^. This could be within a group setting, but we focus on one-to-one moments of interaction. We assume practitioners will be prepared to use their body and voice, but will not (necessarily) have expertise in singing, music, or with any particular instrument.

### Communicative Musicality, Social Timing, and the Kinetic Melody of Play

Playful human interaction has been described as *kinetic melody* (Luria, 1973), as a *dance unfolding* (Stern, 2010), in terms of *primary intersubjectivity* (Trevarthen, 1998; Trevarthen and Delafield-Butt, 2017a) or *Communicative Musicality* (Malloch and Trevarthen, 2009). Neurotypical infant play is an unfolding, real-time, micro-timed orchestration of communicative acts (of movement, sound, and intention). This necessitates a mode of social timing in which both players *continuously communicate near-future states* and *plan communicative acts just-ahead-in-time*.

For us, dynamic *social timing* represents temporal aspects of the ‘dance’ of interaction (Wimpory et al., 2002). It involves intra- and inter-personal levels of cross-modal expression, processing and comprehension that support a deep feeling of connection. Social timing is a key factor in the embodied dynamics of *primary intersubjectivity* (Trevarthen, 1998) defined here as: *the early patterns of affective relating, enabling the sharing of intentions and interest for learning and growth* (Trevarthen, 1998; Trevarthen and Delafield-Butt, 2017a). These patterns are inherently rhythmical. Humans share a pre-position to perceive, move, and interact in temporal, rhythmic ways (Papoušek, 1996; Trevarthen, 1999; Osborne, 2009). There is rhythmicity in the real-time interface between the human body in motion, the neurotypical organization of motor acts, the pattern and impulse generation of biological clocks, and human relational dynamics (Papoušek, 1996; Trevarthen, 1999; Osborne, 2009).

At the heart of intersubjective regulation, embodied rhythmicity enables the coupling of information within, and between, the pre-motor cortices and certain sub-cortical bodies which energize the feeling tones of intentional acts (brain stem, basal ganglia, and limbic structures) (O’Rahilly and Müller, 1994; Holstege et al., 1997; Trevarthen, 1999; Grahn and Brett, 2007; Rizzolatti et al., 2014). “The movement-creating reticular networks and nuclei are intricately combined with the neurochemical systems of emotion. The same activating neurones that select movements and control their energy and smoothness also cause changes in the emotions felt, and the intensity and ‘color’ of consciousness” (Trevarthen, 1999, p. 161). Rhythmicity modulates our movements and colors them with the feeling of momentum, emotion and purpose. It modulates coherent whole-body transformations, enabling the body to express intentions *as one system*. Rhythmicity, “… expresses an integral stream of events created in the whole brain, which conduct separate body parts to targets… synchronizing moves so the effects of separate actions can balance one another and form anticipated sequences and coincidences in space and time, as nearly faultlessly as possible. The gracefulness of all we do depends on it” (Trevarthen, 1999, p. 160).

Social timing encompasses both individual motor timing (intra-personal sensorimotor integration) and social-motor-synchrony (SMS) (temporal alignment of the perceptions, predictions, and motor behavior of two or more people) (Wimpory et al., 2002; Fitzpatrick et al., 2016). SMS relies on predictions generated within the rhythmic flow of interaction and, in turn, enables the furthering of those rhythms. The rhythm of interaction occasionally falls into direct sync, more often feels like two complementary players sharing different parts yet following the same time-frame, and often falls out of sync entirely – needing repair (see section “Good-Enough Social Timing and Learning through Repair” below) (Feldman and Eidelman, 2004; Feldman, 2007a,b).

Through the parameters of Communicative Musicality – *pulse*, *quality*, and *narrative* – we can describe the rhythmic flow of play and come to understand how this flow facilitates the anticipation of an other’s near-future actions, and supports the just-ahead-in-time organization of action with intent (Malloch and Trevarthen, 2009).

*Pulse* is described both in terms of neurobiological time-keeping (the pattern generation of biological oscillators with regularity and momentum) *and* in terms of the related behavior produced: “pulse is the regular succession of discrete behavioral steps through time, representing the “future-creating” process by which a person may anticipate what might happen and when” (Malloch, 2017, p. 65).

*Quality* refers to, “… modulated contours of expression moving through time. These contours can consist of psychoacoustic attributes of vocalizations – timbre, pitch, volume – and/or attributes of direction and intensity of the moving body” (Malloch and Trevarthen, 2009), akin to what Stern named *vitality affects*^8^.

**FIGURE 1:**
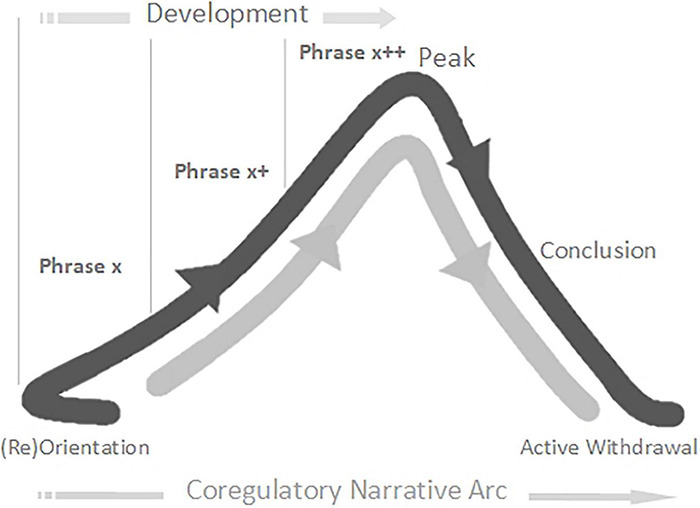
The *Co-regulatory Narrative Arc* of early playful interaction in typical development.

*Narrative* refers to the story-arcs through which we travel together in play, from birth onward, composed in non-verbal rhythms and contours of voice and movement (Malloch and Trevarthen, 2009; Trevarthen and Delafield-Butt, 2013b; Delafield-Butt and Trevarthen, 2015). Each narrative arc is co-created between players, as they share and develop expressive acts, and often follows a flow through discernable stages: Introduction; Development; Climax; Resolution (Malloch and Trevarthen, 2009; Trevarthen and Delafield-Butt, 2013b). The narrative arc helps us get a feel for the flowing build-up of play. Co-regulation is enabled when players travel together in good-enough synchrony through familiar-enough narrative arcs of a particular quality (i.e., flowing in an out of anticipation, vulnerability, and calm) (Schore, 1994; Porges et al., 1996; Porges, 2021; Porges and Daniel, 2021). In emphasis, here we will use the term *co-regulatory narrative arc* as defined by the following model (slightly adjusted from Brazelton et al., 1974): (Re)Orientation; Development; Peak; Conclusion; Proactive Withdrawal (Figure 1). The concept of the narrative arc as a co-regulatory, therapeutic experience has been tested, for work with individuals with autism, within Intensive Interaction (Delafield-Butt et al., 2020), Play Therapy (Daniel, 2019), and Developmental Movement (Daniel, 2017).

Critical to Rhythmic Relating will be the sensitive layering of individually tailored sensory combinations. Different clients are likely to benefit from the parameters of communicative musicality being facilitated in different sensory ways. Importantly then, *pulse*, *quality*, and *narrative* are reported as experientially *amodal* (beyond modal) (Malloch and Trevarthen, 2009). They communicate the *patterned essence* of dynamic happenings, and that essence can be experienced, communicated, and perceived *via any* sensory modality or combination.

## Prediction, Social Timing, Individual Motor Planning, and Social Motor Synchrony in Early Autism

### Autism and Synchrony

Positive measures of synchrony in TD *preverbal interaction* provide developmental correlates for the onset of later interactive, cognitive, and symbolic functions (reviewed by Leclère et al., 2014; Feldman, 2017, 2020; Bell, 2020; and by Wimpory, 2015, regarding the implications for autism). These developmental functions include: later interactive synchrony (at 9 months and 2 years, Feldman et al., 1996); symbolic functioning/social pretense (Harrist et al., 1994; Feldman and Greenbaum, 1997); social-emotional adaptation (Feldman and Eidelman, 2004); empathy (reviewed by Feldman, 2017) and the brain basis of affect-specific empathy (Yaniv et al., 2021); and, later acquisition of language skills (Rohlfing and Nomikou, 2014).

The same developmental functions, when disrupted, are core areas of disability in autism: interactive synchrony (Feldstein et al., 1982; including turn-taking, Lee and Schertz, 2020); symbolic functioning/social pretense (Hobson et al., 2009; Varga, 2011); social-emotional adaptation (Tse et al., 2021); empathy (Koehne et al., 2016; Song et al., 2019; plus careful critical consideration via: Fletcher-Watson and Bird, 2020); later acquisition of language skills (Luyster et al., 2008*)^9^, including pragmatics (overview: Lim, 2018).

Children and adolescents with autism (including high functioning autism, HFA) display reduced or atypical synchrony in conditions rating both *individual motor timing* and SMS (meta-analyses: Baldwin et al., 2021; McNaughton and Redcay, 2020*).

Individual motor timing difficulties in autism (including HFA) involve increased *variability* in individual motor output (Gowen and Hamilton, 2013; Kindregan et al., 2015; Kaur et al., 2018), increased *sensorimotor “noise”* disrupting perceptuomotor integration (Gowen and Hamilton, 2013), and *poor temporal integration of sensorimotor information for efficient prospective motor organization and planning* (Cattaneo et al., 2007; Gowen and Hamilton, 2013; Kaur et al., 2018; Su et al., 2020). It is highly likely these local disturbances in individual motor timing *precipitate* SMS disruption (Mottron et al., 2003; Fitzpatrick et al., 2016; Nebel et al., 2016; Su et al., 2020; meta-analysis: Bloch et al., 2019; conceptual perspective: Trevarthen and Delafield-Butt, 2013a; cognitive implications: Cook, 2016).

For autism-neurotypical interaction, bidirectional SMS appears challenged by these individual motor timing disturbances, principally in terms of the complex *temporal organization* needed for *interactive synchronization* (coupling pendulum task, Fitzpatrick et al., 2016; marching and clapping, Kaur et al., 2018; reach-to-grasp, Su et al., 2020). Interestingly, within these simple-parameter tasks (ibid.), *gross* motor deficit or basic delay in information transmission do not appear to be significant. Although, within the dance of interaction, these movement variability factors can exacerbate differences in expressive quality between individuals with autism and those without (Gallese and Rochat, 2018; Casartelli et al., 2020a,b), creating dissonance or intersubjective incongruence (Trevarthen et al., 2006; Trevarthen and Delafield-Butt, 2013a,^2019^). The degree of SMS disruption evidenced in autism-neurotypical interactions correlates significantly with severity of autism diagnosis (Nebel et al., 2016; Kaur et al., 2018; Su et al., 2020; Overview McNaughton and Redcay, 2020*).

Where parents naturally achieve greater early synchrony with their autistic child with learning disability, this has been found to predict positive child communication outcomes up to 16 years later (Siller and Sigman, 2002, 2008). There is a slowly growing body of empirical evidence suggesting that effective targeting of temporal synchrony is a mediating factor within varied therapeutic interventions with young children with autism (with, and without LD) (Wimpory et al., 1995, 2007; Landa et al., 2011; Pickles et al., 2015; Srinivasan et al., 2015; Dvir et al., 2020; Forti et al., 2020; Griffioen et al., 2020; Whitehouse et al., 2021). Meta-analyses conclude the early primacy of synchrony challenges in autism (including HFA), yet cite the lack of focused therapies (Bloch et al., 2019; McNaughton and Redcay, 2020*; Baldwin et al., 2021).

### Clock Genes and Their Possible Influence in Autism

Timing genes, with multi-level influences across various tides of biological time from circadian rhythms to high-frequency oscillators, are indicated in several genetic studies of autism (usually with co-morbid LD: Wimpory et al., 2002; Nicholas et al., 2007; Nguyen et al., 2010; Neale et al., 2012; Bowton et al., 2014; Yang et al., 2016; Briuglia et al., 2021). It is noteworthy that circadian-associated clock genes can be multifunctional, operating in systems and gene pathways additional to driving the circadian rhythm. For example, the autism-associated clock gene, *RORA* (Nguyen et al., 2010*), harbors causative mutations in certain individuals with HFA or autism plus mild LD (Guissart et al., 2018). This gene is also essential for normal cerebellum development and typical movement (Dussault et al., 1998) and for the development of murine primary somatosensory maps (Vitalis et al., 2018). The role of the clock gene, *per*, in modulating high frequency oscillators in *Drosophila*, concerned with fine motor control and social timing (Kyriacou and Hall, 1980; Beaver and Giebultowicz, 2004), together with findings of genetic association of *PER1* with autism, with and without LD (Nicholas et al., 2007; Neale et al., 2012; Yang et al., 2016), strengthens the notion that clock genes contribute to temporal deficits in autism.

### Autism and Auditory-Temporal Processing

Individuals with autism often display altered auditory temporal processing, including: difficulties detecting duration changes among auditory stimuli (Lepisto et al., 2006); atypical responses to the temporal structure of discrete auditory stimuli (Lepisto et al., 2005, 2006); prolonged latency in unmodulated acoustic startle response (ASR) (Ornitz et al., 1993*); difficulties discriminating timing information between sequential auditory stimuli (Kwakye et al., 2011); delayed latency of evoked potentials in superior temporal gyrus in response to tones of various pitch (Roberts et al., 2010); and difficulties in reproducing auditory stimuli of standardized duration (Szelag et al., 2004).

It is possible that, not only is the neural response to timing information in auditory stimuli atypical for many people with autism, but the timing of the brain’s response itself is *delayed* in response to auditory input (Oram Cardy et al., 2005; Kwakye et al., 2011). These delays may result in a decrease in signal-to-noise ratio of neural signaling for auditory cues, resulting in the autistic experience of sensorimotor “noise” (Brincker and Torres, 2013*; Gowen and Hamilton, 2013) and disturbed time-locking of neural response to discrete sensory events (see Rubenstein and Merzenich’s, 2003, theoretical account). Russo et al. (2009) have demonstrated that the Event Related Potential (ERP) response to auditory speech stimuli, in the absence of background noise, for children with HFA is similar to that *with* background noise for children with TD. This suggests that there may be a degraded response to auditory stimuli at baseline in ASD (Kwakye et al., 2011).

### Autism, Movement Control and Variance

Motoric anomalies in autism (distinct from LD and TD) have been detected at between 3 and 5 months (Esposito et al., 2009) and by approximately 1 year for gait differences (Esposito and Venuti, 2008; Esposito et al., 2011). Subsequent proof-of-concept longitudinal research using wearable sensors has identified reduced motor complexity from as young as 3 months of age and at each 3-monthly time point studied, in two of five genetically high-risk infants later assessed (Wilson et al., 2021). These two infants were the only ones who received a subsequent diagnosis of ASD. Furthermore, the correlation, between motion complexity and ASD-outcome, was stronger than the correlations between motion complexity and outcomes pertaining to adaptive skills and cognitive ability (ibid.).

There are several brain regions involved in pre-motor and motor control which are implicated in autism studies: cortical pre-motor areas [pre-SMA (Puzzo et al., 2010), pre-motor cortex (Silk et al., 2006; Puzzo et al., 2010)], and subcortical areas [basal ganglia (Nayate et al., 2005; Rinehart et al., 2006; Qiu et al., 2010; Estes et al., 2011), brainstem (review: Delafield-Butt and Trevarthen, 2017)]; and cerebellum (review: Courchesne et al., 1994; Akshoomoff et al., 2002; Nayate et al., 2005; Rinehart et al., 2006; Mosconi et al., 2015).

### Autism and Prospective (Just-Ahead-in-Time) Motor Planning

Children with autism often display disrupted prospective motor organization of intentional movement (Mari et al., 2003; Rinehart et al., 2006*; Cattaneo et al., 2007; Fabbri-Destro et al., 2009; Chua et al., 2021). Such “autism motor signatures” can be computationally identified from 2 ½ years of age (Anzulewicz et al., 2016*), and may be detectable from birth. Reduced prospective sensorimotor control of arm movements has been observed in prematurely born infants at-risk for neurodevelopmental disorders (Delafield-Butt et al., 2018). At 3–6 months old, as compared with TD infants, infants with autism showed significantly less anticipatory mouth opening in response to an approaching spoon at feeding times (Brisson et al., 2012*). A series of retrospective home-video studies have documented evidence that infants with autism (younger than 12 months) display a lack of organized anticipatory social behaviors (Adrien et al., 1991s*; Osterling and Dawson, 1994; Baranek, 1999*; Maestro et al., 2001; Trevarthen and Daniel, 2005; Brisson et al., 2014).

Moving with organized intent involves the just-ahead-in-time generation of a spatiotemporally coherent motor ‘image’, organized to achieve movement with efficient purpose (Lee, 2009). This prospective, intentional motor control is operative from before birth from the 2nd trimester (Delafield-Butt and Gangopadhyay, 2013). Its intentions first reach into the imminent future of just 1–2 s, but in human development this soon extends to enable goals that are many tens of seconds, minutes, even hours, days or years into the future (Trevarthen and Delafield-Butt, 2017b).

For people with autism, this essential motor image may be disrupted in space or time, due to disturbed temporal integration of multimodal information. For example, in a precision grip task, two *temporal* variables (load force onset latency and time to peak grip force) and two *force* variables were used to differentiate children with ASD and TD children (David et al., 2012). Children with ASD presented with significant motor coordination challenges *only* on the temporal variables. The researchers concluded, “… that subtle problems in the timing of motor actions, possibly related to maturational delays in anticipatory feed-forward mechanisms, may underlie some motor deficits reported in children with ASD” (ibid).

In a reach-to-grasp task, autistic individuals did not rhythmically coordinate the reaching of the arm and the opening of the fingers in a fluid intentional flow – instead they performed one act and *then* the other separately (Mari et al., 2003). In contrast, TD children coordinated intentional sequences of arm and hand actions fluently in “pre-reaching” and gesturing from early infancy, to achieve coherent goals distal in time and action space (von Hofsten, 1984).

Children with autism displayed motor impairment without any deficits in proprioception, during a simple elbow flex-extend task (Fuentes et al., 2011). These findings may indicate that proprioceptor sensors are neither hyper-, nor hypo-sensitive in individuals with autism, rather it may be that temporal integration of proprioceptive information with other sensory input is disturbed (ibid.).

Prospective movement involves the just-ahead-in-time arrangement of single motor acts into action-chains, organized with advance respect to the movement requirements of *the intended goal* (Fogassi et al., 2005; Bonini et al., 2011; Rizzolatti et al., 2014; Rizzolatti and Sinigaglia, 2016). Experiments with monkeys have demonstrated that single motor acts will be organized by markedly different pre-motor and parietal neuron activation, when this act is part of action-chains that have different *goals* [e.g., grasping food for eating (Chain A) vs. grasping food for placing (Chain B)] (Fogassi et al., 2005; Bonini et al., 2011). The first motor act in a functional chain is organized and tagged with regards to subsequent acts, this tag being the neural handle to instigate the whole action-chain. For instance, in Chain A, on activation of the initial motor act (reach-to-grasp), neurons will fire simultaneously which facilitate pre-emptory mouth opening. The same initial motor act (tagged differently in Chain B) will not pre-empt mouth opening.

Across various tasks, including a version of the above experiment adjusted to assess the Electromyography (EMG) activity of the mouth-opening MH muscle in human children, Cattaneo et al. (2007) demonstrated that goal-specific action-chaining exists for TD infants. While for children with autism, in the same tasks, goal-specific action-chaining is significantly impaired: “…for TD children, the EMG activity of the MH muscle started to increase several hundred milliseconds *before* the hand grasped the food. It continued to increase during actual grasping, and, as expected, it reached its peak when the individual started to open the mouth. The behavior of the MH muscle found in children with autism was strikingly different. In this group, no activity increase was found during the entire reaching and grasping phases. The muscle became active *only* during the bringing-to-the-mouth phase” (Cattaneo et al., 2007, p. 17827).

Moving in interactive synchrony with another person involves just-ahead-in-time understanding of their intention, which advance-informs the generation of our own spatiotemporally coherent motor image. Goal-oriented action-chaining, coupled with “action-constrained” mirror neuron activation on observation of another’s initial motor act, plays a significant role in the anticipation of another’s intention (Fogassi et al., 2005; Cattaneo et al., 2007). “By activating a specific action chain from its very outset, this mechanism allows the observer to have an internal copy of the whole action before its execution, thus enabling them to understand directly the agent’s (*functional*) intention” (Cattaneo et al., 2007, p. 17825, italics added). TD infants experience the intentions of others experientially and as gestalts, *prior* to understanding them cognitively (Cattaneo et al., 2007). Children with ASD do not (Cattaneo et al., 2007; Boria et al., 2009). HFA children may understand the intentions of others cognitively but lack the mechanism for understanding them experientially (Cattaneo et al., 2007).

It is significant to note here, that the accuracy of mirror neuron information is defined by the accuracy of the internal motor image onto which it maps. The former can only be as good as the latter. As such, it is highly likely that mirror neuron dysfunction is secondary to individual motor planning disturbance in autism, across goal-oriented action-chaining (Cattaneo et al., 2007).

### Autism and Vitality-Forms

There are four dimensions to understanding and pre-empting another person’s actions. There is the practical *what* of an action (for instance, *she moves her arm, grasps a cup*). Children with autism, as compared to TD children, demonstrate no difficulties in interpreting the *what* (Boria et al., 2009). Then there are three dimensions which communicate different aspects of intention: the *how*; the *emotion*; and the *goal*.

The *how* dimension is communicated by *vitality-forms*^10^, a term which refers to the affective energetic vector of a particular (potentially communicative) act: a waving hand could be vigorous (possibly welcoming, possibly warning) or gentle (possibly kind, possibly hesitant). “By recognizing the vitality-form of an action, one can appraise the affective/cognitive state of an agent as well as his/her relationship with the action recipient” (Di Cesare et al., 2014, p. 951).

Children with ASD, as compared with TD children, demonstrate significant differences in vitality-form expression (Casartelli et al., 2020a), and challenges in recognition of TD vitality-forms (imitation studies – Hobson and Lee, 1999; similarity judgments – Rochat et al., 2013; immediate evaluations – Di Cesare et al., 2017a).

### Temporal Integration of Multimodal Information for Motor Planning: The Neurobiology of a Core Feature in Autism

Recent functional near-infrared spectroscopy (fNIRS) data suggest that, during tasks designed to elicit SMS, children with high-functioning autism (as compared with TD children) display hypoactivation in the *middle and inferior frontal gyri* (MIFG) as well as *middle and superior temporal gyri* (MSTG), while showing hyperactivation in the *inferior parietal cortices/lobule* (IPL) (Su et al., 2020). Here, IPL hyperactivation suggests dysfunction^11^. The IPL is an association cortex, where multimodal information becomes integrated for the tagging of motor acts (Mountcastle et al., 1975; Yokochi et al., 2003; Fogassi et al., 2005).

Goal-oriented action-chaining engages a network of cortical regions [pre-motor cortex, supplementary motor area (SMA), and pre-SMA] which loop information *via* critical subcortical bodies (including the basal ganglia and cerebellum) and route information *through the IPL* (Fogassi et al., 2005; Bonini et al., 2011; Rizzolatti et al., 2014; Rizzolatti and Sinigaglia, 2016). Vitality-form expression (Di Cesare et al., 2015, 2018), vitality-form recognition (Di Cesare et al., 2014, 2016), and vitality-form mental simulation (Di Cesare et al., 2015) involve activation of the dorso-central insula^12^, a pathway which links sensorimotor cortical areas with the limbic hippocampus, including the IPL and MFIG (Di Cesare et al., 2017a).

Crucially, the IPL is *the* region of functional overlap between networks responsible for SMS, goal-oriented action-chaining, and vitality-form recognition. Concurrent dysfunction in these networks, overlapping at the IPL, gives significant weight to the hypothesis that autism involves primary dysfunction in the temporal integration of multimodal information for motor planning.

Yang and Hofmann’s (2016) meta-analysis of functional magnetic resonance imaging (fMRI) studies assessing disturbance in imitation (in response to observed action), concludes that the strongest significant effects, distinguishing TD-ASD populations, were exhibited in the anterior IPL.

### What Does Disrupted Bidirectional Social-Motor-Synchrony Look Like in Play?

**FIGURE 2:**
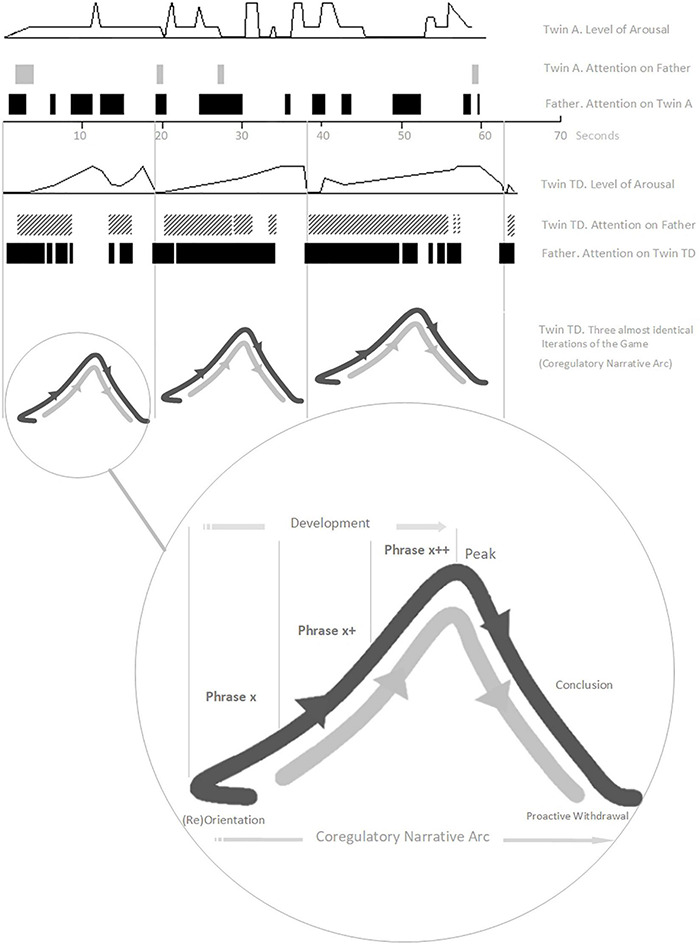
The “Monster Belly Blow Game” (Twin TD, Typically Developing; Twin A = with Autism). The *Arousal* and *Attention* variables are aggregate scales drawn from combining relevant interactive behaviors from a tailored list developed for this micro-analysis (please see Trevarthen and Daniel, 2005, for details). Here, *Arousal* corresponds to Social Engagement System arousal. Re-presentation of findings – with authors’ permission.

Here, we illustrate several of our working concepts with an extended example. In a study involving micro-analysis of home video tapes of two infant monozygotic twins, early interaction differences were described between a typically developing girl (Twin TD) and her twin sister with autism^13^ (Twin A, 11 months-old, pre-diagnosis), both playing a game (“*The Monster Belly Blow Game”*) with their father (Trevarthen and Daniel, 2005). The video analysis was done retrospectively, after Twin A received her diagnosis at 3 years old. The recorded interaction with Twin TD involved three, almost identical iterations of the game (Figure 2).

Each game (described by a co-regulatory narrative arc) begins with a moment of orientation between Twin TD and her father. In most iterations this is described by the father initiating and Twin TD close-on-immediately orientating with eye-contact and increased motor tonus. The game then develops, with the father building playful anticipation through three to four iterations of a particular *extended moment* of social stimulation – “*the monster arrives*” (combining looming and vocal contours of rising volume and pitch).

We consider such extended moments to be basic units of interaction – building blocks of a developing game, 2–8 s in duration^14^, which we are calling *phrases*. Phrases of play can vary greatly in their look and feel. Sometimes a phrase will have its own contour, itself constituting a small *narrative arc*, which becomes nested into the larger co-regulatory narrative arc of the game. Many early game structures include several iterations of such phrases strung together in series or combination, nested in the *development* stage of the game (Figure 1). Often, each iteration involves a step-up in *playful anticipation*, an energy which looks and feels very different depending on the mood of play: the quiet wonderment of a whispered suggestion; the nervous joy in the not-quite-sureness of “hide-n-seek”; the raw joyous anticipation of “peek-a-boo”; or the angry edges of challenging or rough-and-tumble play. The joy of this anticipation, even more than its culmination in a peak, impels the child’s expression of, “AGAIN!”. The repetitious layering and rhythm of phrases supports fluid synchrony of attention, anticipation, and action – providing each player with information, impulse, and momentum about the near-future actions of the other.

Back to the Game. Twin TD remains transfixed and displays heightening anticipation throughout. Each game then reaches its peak with a “*monster belly blow*” from the father (physical stimulation – blowing on and tickling belly – with an accompanying “growl” of high volume, low pitch, and playful gravelly timbre). A period of withdrawal and self-regulation then occurs as Twin TD breaks eye-contact and releases motor tonus. This is a proactive stage, however, as Twin TD is waiting for re-initiation from her father and responds close-on-immediately when he engages. The second game (a new narrative arc) begins.

Twin A displays asynchrony in attention, behavior, and motor tonus, and her behavior lacks any coherent build-up of arousal or anticipation (Figure 2). The attempt at play with Twin A lacks structured phrases or a narrative arc – it is not a game at all. Twin A’s behavioral style does not promote or modulate, in her father, the typical impulse to engage with graceful rhythmicity. Already (remember the twins are only 11 months old, way in advance of diagnosis, with the parents retrospectively reporting no concerns) the father has tacitly and completely adapted his interaction style. Wanting, naturally, to make his daughter happy, the father has dropped all attempts at social build-up with Twin A reverting, instead, to frequent moments of purely physical stimulation (the *“monster belly blow”).* Sadly, this natural adaptation will likely perpetuate asynchrony and asocial behavior in Twin A as she develops. The father, like many parents and practitioners, needed encouragement and therapeutic tools with which to tailor specialized attempts to connect with his daughter with autism.

### Summarizing Working Assumptions (1)

Support for social timing should facilitate perceptual discrimination, timing, and contiguity of sensory input (for instance, reducing audio “noise” and latency). It should facilitate the temporal integration of multimodal information for just-ahead-in-time motor planning, and the just-ahead-in-time prediction of an other’s communicative acts. It should facilitate the rhythm and phrasing of play. It should facilitate alignment of the movement/sound patterns of client and practitioner into good enough synchrony. Co-regulatory SMS should include a wide range of synchronous experience and involve a variety of narrative arcs in which partners travel together through different types and levels of arousal and back to calm.

## Rhythmic Relating for Autism: Essential Parameters

### Good-Enough Social Timing and Learning Through Repair

Social timing and reciprocity are by no means seamless in TD infant interaction. Playful partners flow in and out of levels of synchrony. Sometimes partners share *Simultaneity*^15^, (traveling alongside each other, directly in-sync), experiencing concurrent occurrences of specific behaviors in parent and child, such as the co-occurrence of social gaze, vocalizing together, the matching of arousal level, or the coordination of parent affectionate touch with infant social gaze (Feldman and Eidelman, 2004). Sometimes partners share *Complementarity* (the behavior of one partner complimenting the other’s in timing and quality, while relating to the same pulse). In *complementarity*, behaviors often coalesce into repetitive “configurations”, creating early rhythmic structures with inherent temporal expectations (Feldman, 2007b); expectations which can then be challenged and extended in playful *teasing-around-timing* (e.g., peepbo” vs. “pee….p-bo!” vs. “pe-ee……..p-bo!”) (Wimpory, 1995, 2015). This is particularly a feature of more sophisticated (bidirectional) *mutual synchrony* whereby each partner shares a more influential role (Feldman, 2007a,b). Such playful reciprocal “double-take” of expectations, during preverbal play with, and without objects, may facilitate appreciation of the “double meanings” required for symbolic play (Trevarthen and Logotheti, 1987; Reddy, 1991; Wimpory, 1995; Wimpory and Gwilym, 2019). Sometimes, partners are out of sync. Tronick and Cohn (1989) found that infants regularly experience interactive miscoordination, yet mismatch is typically repaired close-on-instantaneously (review: Tronick, 1989). “This constant oscillation between momentary miscoordination and interactive repair marks the essence of human dialogue, to which infants are sensitized in their earliest interactions” (Feldman, 2007b, p. 341) It is, in part, through the developing ability to recover synchrony when it goes off track, that TD infants may learn robust, flexible socio-communicative skills. For people with Autism^(Therapeutic Needs)^, of course, the initial priority is enabling good-enough synchrony and helping to repair it where needed.

### Acclimatization, Simplicity and Sameness

People with Autism^(Therapeutic Needs)^ experience pervasive sensory disruption and fracturing. When this experience is acute, most often accompanied by heightened arousal levels, interaction is unfeasible. As practitioners we can help with a structured environment: minimal and consistent across all sessions; clean; low light levels; low levels of ambient sound; minimize smells; private (no disturbances); unconditional opportunities for time-out and rest. Long-term consistency of practitioner is also essential. A period of acclimatization may be a crucial precursor to potential playful interaction. In this period, practitioners should prioritize client-led sensitivity; sensitively mirroring while adding nothing; maintaining low levels of arousal; observing the client’s needs, including the need for short-duration engagement, time-out and rest. As practitioners, we can be sensitive to when trust and simplicity become established – and feel carefully for those moments when our tentative input becomes feasible rather than damaging.

### Engaging Existing Heterogeneous Movement and Sound

Torres et al. (2013) helped children with autism gain improved fine motor control of their hand movements. They used a co-adaptive child-computer interface (visuo-spatial and auditory) to augment each child’s spontaneous movement experience and substitute corrupted kinesthetic information. The aim was to give existing heterogenous movement patterns a chance, seeing these patterns as meaningful adaptations with personal momentum and rhythmicity. The study (and the trust) worked. “… This new concept demonstrates that individuals with autism do have spontaneous sensory-motor adaptive capabilities. When led to their self-discovery, these patterns of spontaneous behavioral variability (SBV) morph into more predictive and reliable intentional actions. These can unlock and enhance exploratory behavior and autonomy in the individual with autism.”

### Engaging Initial Loops of Behavior

Infants with Autism^(Therapeutic Needs)^ often appear to channel the potential momentum of social engagement into interactions with the non-human environment (Trevarthen et al., 1996; Maestro et al., 2005). Disconnected from interaction, the infant’s rhythmicity often appears in loops of repetitive behavior: movements and/or sounds [often referred to colloquially as “stimming” (Bakan, 2014)]; relational patterns; or configurations of object-play. The loop is a self-completing pattern, with impulse and energy within either the beat pattern of repetitive movement or sound, or the discrete behavioral steps in a section of object-play. Each beat/step is essential to the pulse of the loop, to its momentum and sense of completeness. If present, we, as practitioner, can piggy-back on that pulse. If looping regularity is not apparent, we can find ways of integrating a pulse and moving from there.

### Recruiting Auditory Beat as a Tool to Facilitate Social-Motor-Synchrony? A Neurobiological Perspective

In musical experience^16^, the presence of a beat enables the perception of rhythm and compels movement (Grahn and Brett, 2007; Grahn, 2012; Levitin et al., 2018). Why is this?

Pre-motor organization engages a network of cortical regions [pre-motor cortex, supplementary motor area (SMA), and pre-SMA], which loop information *via* critical subcortical bodies (including the basal ganglia, brainstem and cerebellum), and route information through the IPL (Fogassi et al., 2005; Bonini et al., 2011; Rizzolatti et al., 2014; Rizzolatti and Sinigaglia, 2016).

The SMA, basal ganglia, and the cerebellum comprise an, “extended cortico-subcortico-cortical functional network providing specific timing and entrainment sensitivities” (Nozaradan et al., 2017, p. 156), in the processing of auditory rhythm – the beat perception/generation network. Biological oscillators in these respective regions provide overlapping yet different time-signature ranges, and therefore differing functions, which complement each other in the full creation of rhythmic experience (Ivry and Hazeltine, 1995; Ivry, 1996; Grahn and Brett, 2007; Grahn, 2009; Nozaradan et al., 2017).

This beat perception/generation network shares significant structural and functional overlap with the pre-motor organization network. Oscillatory function within the pre-SMA, the SMA, and the basal ganglia in particular is integral to both beat generation *and* pre-motor organization (Grahn and Brett, 2007). The basal ganglia and the pre-SMA/SMA are richly connected through striato–thalamo–cortical loops (Alexander et al., 1992; Inase and Tanji, 1994) and are involved in the prospective timing of future movements (Alexander et al., 1992; Rao et al., 1997; Sardo et al., 2000; Tan et al., 2009). “A role for the basal ganglia and SMAs in beat induction is consistent with their involvement in motor prediction (the spontaneous response to hearing a beat is often to move at the time when the next beat is predicted)” (Grahn and Brett, 2007, p. 902).

The potential for beat-based SMS facilitation relies, of course, on intact beat-perception. Across simple and complex meter conditions, pre-SMA, basal ganglia, and cerebellar dysfunction in autism appears to be functionally specific, leaving beat-perception largely intact (DePape et al., 2012). The anatomically and functionally specific nature of basal ganglia dysfunction in particular (Rinehart et al., 2006; Qiu et al., 2010) may likely allow for a window of beat-based support.

### Schweep Schwop Not Tick Tock: Information-Rich Rhythm Helps Individuals With Autism to Predict

Knight et al. (2020) presented moments of potential *prediction error* (when a sound occurred earlier than expected in a regular series) to individuals with ASD (recruited *via* a multi-level assessment to factor out possible confounding variables; 6–21 years.). As discussed in section “Prediction, social timing, individual motor planning, and social motor synchrony in early autism,” hypersensitivities, sensorimotor “noise,” relative latency, and disrupted multimodal integration all have the potential to set people apart in time and disable just-ahead-in-time prediction. In section “Autism and Auditory-Temporal Processing” in particular, we outlined the case for predictive delay and/or asynchrony in autism in response to isolated sonic events. However, when presented *within simple or complex rhythms*, prediction error was completely absent – no difference was found in ERP response patterns between individuals with ASD and neurotypical controls (Knight et al., 2020).

Importantly here, our concept of rhythm is *information-rich*; full of temporal and non-temporal cues (potentially multimodal) to guide participants to on and off-beat moments in time, intention, and action. This is crucially different from the clinical tick-tock of the metronome. A meta-analysis of “tapping studies” (Yoo and Yoon, 2019), included six studies which examine unilateral (simple) movement response patterns^17^ (i.e., basic tapping) as a synchronization response to “auditory stimuli”. All but one of these, result in ASD subjects performing worse at synchronization than TD controls (as expected, see sections “Autism and Synchrony” and “Autism and Auditory-Temporal Processing”). One study, Tryfon et al. (2017), was exceptional – demonstrating no significant difference between ASD and TD performance, and concluding non-verbal rhythm synchronization is intact for children with ASD. On examination, all of the preceding five studies presented auditory stimuli as a “paced beat” (a straight tick-tock generated by computer or metronome). Tryfon et al. (2017) presented audio recordings of woodblock *rhythms* (complex, medium, and simple meters). We suggest this stand-out feature, in relation to the stand-out result, is not coincidence.

Yoo and Kim (2018) explored the impact of dyadic drum playing on children with HFA, concluding that the presence of rhythmic cueing and sensitive tempo adjustment correlated with improved measures of social skills. Recruiting this information-rich rhythmical interaction as an intervention over time, Yoo and Kim (2018) facilitated synchrony between children with ASD and neurotypical partners. After the intervention, participants showed decreased asynchrony when tapping with a partner at adjusted tempi, and showed greater engagement in joint attention and action.

Forti et al. (2020) developed a synchrony training program in which children with ASD were shown a progression of meaningless arm movements, with associated melodic/rhythmic scaffolding, and were asked to imitate the movements. Over 6 weeks, the children improved across increasingly difficult task variants in measures of synchrony and imitation (ibid.). Srinivasan et al. (2015) demonstrated that, over 8 weeks, children with ASD benefited from a rhythm-based movement intervention, displaying improvements in body coordination, imitation/praxis, and interactive synchrony.

In a study designed to compare music versus non-music interventions, ASD groups were assessed before and after on measures of social communication and resting-state functional connectivity of fronto-temporal brain networks (Sharda et al., 2018). Over 8 – 12 weeks the music intervention group (where improvisational approaches, involving song and rhythmic scaffolding, were used to target social communication and sensorimotor integration) scored significantly higher on a measure of pragmatic communication (*P* = 0.01). Significantly (*P* < 0.00001), post-intervention resting-state brain connectivity was *lower* between auditory and visual regions in the music compared to the non-music groups, *showing a reduction in disruptive over-connectivity* (known to be prevalent in autism, ibid.; + see Courchesne et al., 2001, 2003).

The human ASR is a neurophysiologically fast and direct response to certain sudden, unexpected auditory stimuli. ASR latency – the time from presentation of the startling stimulus until neural response – provides an index of neural processing speed. As discussed in section “Autism and Auditory-Temporal Processing,” individuals with autism demonstrate prolonged unmodulated ASR latency as compared with age-matched TD controls (though without controls for LD, Ornitz et al., 1993*). Understanding the conditions which modulate latency is important for us here as, if we can minimize relative TD-autism latency difference, we can improve alignment for synchrony. When startle stimuli were presented with pre-stimulation and/or with habituation^18^, latency differences and auditory hypersensitivities (shown *via* ASR amplitude) became non-significant (ibid.). Clearly, predictive information – through context and familiarity – matters.

Rhythmic Relating will build on the pulse inherent in the client’s movement, sound, or object-play and augment it with clarifying qualities and tailored multimodal cues. This client-centered rhythm will provide a flow of predictive information and compelling pulse – clarifying the practitioner’s communication and providing a framework to facilitate sensory contiguity, discernment, prediction and just-ahead-in-time planning.

### Recruiting Acoustic Brain-Stem *Turbulence*: Evolutionary Sounds That Move Us

Recently an innovative computational approach to the automatic categorization of music (X-System) has proved successful in predicting emotional, arousal, and mood responses to music (Sice et al., 2020). Certain specific psychoacoustic qualities produce extremely direct, evolutionarily pertinent responses in humans. These include *ASR-stimuli* and *acoustic activation contours*. Sice et al. (2020) have used the term *brain stem turbulence* to describe these sounds, with reference to the degree to which these sounds constantly change in ways which activate and move us.

The ASR operates along a pathway leading directly from the cochlea, along cranial (auditory) nerve VIII by way of the lateral lemniscus, to the caudate reticular nucleus. From here, there are descending projections to spinal and limb motor neurons, provoking the “jump” or “blink” effect (Frankland et al., 1997; Osborne, 2009).

*Acoustic activation contours* are evolutionarily significant sounds indicative of the positioning and movement of the human body in space and time (from sudden approach, to slowly moving away). This may extend from separation cries (Panksepp, 2003), or the hissing of snakes (Erlich et al., 2013), to rapidly approaching sounds, glides, falling, fast crescendos, bursts of sound and the like. It is very likely that these sounds are recognized by innate systems early in auditory pathways (Erlich et al., 2013; re: the Inferior Colliculus, Jorris et al., 2004; Sivaramakrishnan et al., 2004). There is clear evidence of these pathways ascending to emotional systems (Heldt and Falls, 2003), as well as a descending, emotional “feedback” pathway from the amygdala (Marsh et al., 2002).

Music plays with the use of turbulence, specifically taking the “dangerous” edge of activation and, through context, timing, and expectation, leveraging that energy for joy, wonderment, and anticipation (Osborne, 2020). Moments of *relative acoustic startle* can provide defining beats and turning points. *Acoustic activation contours* can, individually, stimulate changes in mood and energy, and, used as repeating rhythmical structures, they can define the mood of extended moments or of a whole piece of music.

Turbulence compels response through movement. Turbulence is neurophysiologically direct vitality communicated instantaneously *via* sound. *Sensitive* use of turbulence may provide people with autism with a palette of sounds which represents their *most-direct* audio experience (relatively decreased latency and increased signal-to-noise ratio). This *sensitive* use will include tailored multimodal experiences, time given for familiarity and habituation, and modulated volume within an information-rich rhythm. Most clients with Autism^(Therapeutic Needs)^ will find loudness intolerable (stimuli > 80 dB – which is akin to shouting, twice as loud as conversation) (Khalfa et al., 2004), and some may be hypersensitive to particularly high-pitched sounds at normal-to-mid-range volumes (Rosenhall et al., 1999; Takahashi et al., 2014, 2016). For many clients then, we could start quietly, experimenting with *relative acoustic startle* and low-volume *acoustic activation contours*. As such, we may be able to use turbulent rhythmic structures (accents and contours) to add guiding information and energized pulse into interaction with a client with Autism^(Therapeutic Needs)^.

Turbulence may also help us share emotion, mood modulation, and co-regulation. Akin to its use in music, when turbulence is presented sensitively within interactive social rhythms (defined by structure, predictability and safety – i.e., the absence of threat) the mobilization (fight/flight) potential of turbulence is likely to become the stuff of joy, anticipation, and play (Porges, 2021; Porges and Daniel, 2021).

Here, we introduce the concept of a *tonescape*: a “landscape” of interactive possibilities, spanning a wide range of modulated turbulence and synchrony; a landscape full with a variety of co-regulatory narrative arcs, leading partners in and out of varying levels of arousal and emotional tone. The tonescape can bring opportunities for small-step co-regulation and layered SI. The range of modulated turbulence in the tonescape, reaches from the poignance of peace shared, to the wonderment of subtle variation (playful, fluid, unexpected, an emotional “hide-n-seek”), to the raw joyous anticipation of “peek-a-boo.”

### The Versatility of Activation Contours

Activation contours are short expressions of quality which stimulate an (inter)active state change. They are single events, often multimodal, communicating vectors of intention in movement and sound, containing “…the felt experience of force… with a temporal contour and a sense of aliveness, of going somewhere” (Stern, 2010, p. 3). Activation contours can be the building blocks of a developing game – shared experiences in repetition or combination. They can be tools for the embodied reflection of vitality. They can be recruited as stand-alone events, promoting interest and motivation if things feel stuck. And they can also be integral to rhythmic synchrony scaffolding (see later) as up-beat guides to an on-beat shared moment.

Activation contours come in a huge variety and subtlety of types. Here we describe seven of them – possibly the most prevalent in interaction^19^, and those which we have found most useful to consider in practice:

*Up-Swish* – an upwards inflection guiding toward a moment in time, space, expressed energy, and emotional tone.

*Down-Swoosh* – a downwards inflection guiding toward a moment in time, space, expressed energy, and emotional tone.

*Stretch* – a consciously elongated up-swish or down-swoosh.

*Burst* – an instantaneous, exploding energy often in an outward, interactive vector.

*Quick-fade* – the opposite of the burst, an instantaneous imploding, withdrawing vector.

*Waver* – a wavering vector in either an up-swish, down-swoosh, or an even plane.

*Pulse* – a pulsing vector in in either an up-swish, down-swoosh, or an even plane.

Activation contours can be expressed in any sensory modality or combination of modalities. When envisioning combination possibilities, we have found the following constructs useful (some single-, some multi-modality):

*Volume* – volume change (including silence).

*Proximity* – positional change (relative to the other player).

*Embodiment* – whole-body, postural change.

*Intensity* – change in the level of energy invested.

*Pitch* – audio pitch change.

*Timbre* – vocal emotional-tone change.

The “*Monster belly blow*” game (section “What Does Disrupted Bidirectional Social-Motor-Synchrony Look Like in Play?”) involved the father using a rising vocal inflection (Up-swish in volume, pitch, timbre) whilst looming in toward his daughter (Up-swish in: proximity), then a falling vocal inflection (Down-swoosh in volume, pitch, timbre) whilst looming away (Down-swoosh in proximity), and the “*Belly blow*” (Burst in volume, intensity, and timbre). Other examples are, an energetic star jump (Burst in embodiment and intensity), pulling a client along a smooth floor, cradled in a blanket, with a sideways waggle [Stretch (with interspersed Waver) in proximity, embodiment, intensity] bouncing with a client on a trampoline [Pulse (vestibular) in intensity, embodiment].

### Parameters for Rhythmic Synchrony Scaffolding: Lock-on Beats

Here we introduce the concept of *Rhythmic Synchrony Scaffolding*: the use of rhythm (in any modality or combination) to match, accent, cue, augment, and develop the client’s pulse in movement and sound.

Within music, beats can be easy to perceive, very difficult to perceive, or overtly non-existent. For us, a *lock-on beat* (useful in scaffolding rhythmic experience) is one that maximizes the properties which promote perceptual ease. These properties can be temporal or non-temporal.

The *temporal* properties of a rhythm can induce the spontaneous *feeling* of a beat (Brochard et al., 2003; Grahn and Brett, 2007). For a lock-on beat, it is helpful to keep a regular pattern with a simple meter, i.e., one with short duration intervals, and simple integer ratios (Essens and Povel, 1985; Sakai et al., 1999; Grahn and Brett, 2007). Simple meter patterns, as opposed to complex ones, have been shown to improve synchronization dynamics (Patel et al., 2005). In western music tradition, all time signatures (or meters) are constructed in patterns of 2 and 3 s. Our simplest meters are: 2/4 [evenly accented; defined by a march; example, the Imperial March (Darth Vader theme) in Star Wars]; 3/4 (accented, strong–weak–weak, strong–weak–weak; exemplified by a waltz); 4/4 (accented, One-and-Two-and, One-and-Two-and; examples: the straight “money-beat” which opens Michael Jackson’s *Billie Jean*, or defines Mozart’s, *“A Little Night Music”* and Pachelbel’s *Canon in D major)*.

In beat perception, the basal ganglia-cerebellum partnership displays different patterns of activation on attempted perception of simple or complex meters (Grahn and Brett, 2007; Grahn, 2009; Nozaradan et al., 2017). Using a lock-on beat will engage the basal-ganglia/pre-motor-area relationship, as: “…functional connectivity between part of the basal ganglia *(the putamen)* and cortical motor areas *(the pre-motor and SMA)* is higher during perception of beat rhythms compared to non-beat rhythms” (Grahn, 2009, p. 35, italics added).

Lock-on beats will also simplify cerebellar processing demands and limit the demands of beat generation on the basal ganglia. In rhythm tracking studies involving patients with brain lesions, “… for cerebellar patients… *negative effects were*… specific to the rhythm played at a fast tempo, which places high demands on the temporally precise encoding of events. In contrast, basal ganglia patients showed more heterogeneous responses at beat frequency specifically for the most complex rhythm, which requires more internal generation of the beat” (Nozaradan et al., 2017, p. 156, italics added).

We can also simplify lock-on through choice of periodicity. Many potential rhythms have several levels of periodicity present. For example, in “twinkle, twinkle, little star,” one can tap regularly to every syllable, every other syllable, or every fourth syllable, and still be synchronized to the music (Drake et al., 2000). Initially, we can choose to accent a well-spaced level of periodicity (for instance, the fourth syllable in the above example).

The temporal accents present in certain rhythmic patterns can act as just-ahead-in-time guides, allowing players to land on a moment of emphasis. The feel of this is “the act of raising or lifting,” followed by “setting down” – like lifting a foot before making a step then placing it down with precision. The raising acts as an upbeat, anticipating and guiding the on-beat. In prosody, this is well exemplified by the iambic pentameter with an even pulse: da-Daah, da-Daah, da-Daah, da-Daah… In music, the “da-” becomes the upbeat guide to the “Daah.”

In terms of *non-temporal* support, focal beats within a pattern can be accented with *intensity accents* (Grahn and Brett, 2007). These are single beats, emphasized by a change of intensity in pitch, volume, and/or timbre. Intensity accents can make impacting use of *relative acoustic startle*.

*Activation contours*, with their turbulent, directional energy, can help guide a player to the beat, with just-ahead-in-time advance warning. Guiding a movement toward an intended goal (moment in time; point in space; specific purpose) involves such motor prediction. TD individuals use perceptual force-time curves (with a felt sense of expected time-to-closure) to organize the effective use of force in actions, and to couple these actions with the actions of another (Lee, 1998; Rousanoglou and Boudolos, 2006; Delafield-Butt et al., 2018). In an arm-extension (for example, in expressive gesture, reach-to-grasp, or tap-to-a-beat), the motor image is a force-time curve of energetic enervation: rising on initiation, increasing to reach, falling in expectation to land on point-of-contact, or body-space goal with intention-specific appropriate force (Lee, 2009; Delafield-Butt et al., 2018). We are proposing that activation contours can serve as guides for perceptual-motor force-time curves, helping us land on beat and act in synchronous time-scales (e.g., Schögler et al., 2017).

### Facilitating Quality – The Experience of Vitality

Di Cesare et al. (2017a) have shown that children with HFA have difficulties in perceiving vitality-form differences between two contiguous stimuli (smallest change detected at >100 ms apart). This suggests that, “during action observation, children with ASD need greater stimuli variations than TD children to detect their differences in terms of vitality forms” (ibid. p8). We can support clarity in contiguity through clear isolated communicative acts. Children with autism can often recognize extreme vitality, while lacking distinction of the more nuanced vitality-forms characteristic of everyday interaction (Di Cesare et al., 2017a). Playful interaction gives us the platform to use big, distinct gestures when needed – to initiate and to clarify – and then to build toward sharing more nuanced actions.

Any one vitality-form can be recognized *via* either *visual* or *auditory* expression (Di Cesare et al., 2017b,^2018^). Indeed, Di Cesare et al. (2017a) have concluded, “…it may be plausible that visual information is not sufficient for children with ASD to encode vitality forms correctly and that the use of alternative (*additional*) perceptual information may help vitality form perception” (p. 8, italics added).

Dependent on the client’s level of language comprehension, we can support vitality recognition through explicit labeling of the other contextual intention-dimensions: *goal* and *emotion*. We can verbalize what we are doing and our goal; we can verbalize what the client is doing (using their name in third-person) and their goal, if apparent. We can verbalize our emotions; we can verbalize the client’s gross emotional state (happy, sad, angry, excited), if apparent.

A recent study by Casartelli et al. (2020a,b) focuses on a bidirectional approach to motor dissimilarity in social contexts. Emphasized here, is the fact that TD adults demonstrate deficits in recognition of ASD vitality-forms even after information feedback (Casartelli et al., 2020b). This bidirectional finding suggests that we should avoid our neurotypical-centric interpretations of vitality-forms and, until we have spent time tuning in to our client’s expressions, we should begin with simple mirroring, observation, and trust-building.

### Facilitating Quality – The Psychoacoustic Attributes of Vocalizations

Using structural magnetic resonance imaging, Lai et al. (2012*) found that neuroanatomical systems that process speech and song are more effectively engaged by song than by speech, for children with ASD. We can use a melodic “story-teller’s” voice (light and playful variation in pitch, timbre, volume, overall mood tone – a conscious avoidance of monotone) and/or we can literally sing our communication.

As is the case for short-interval parsing for ease of lock-on in beat perception, short spoken units support ease of rhythmic parsing in language comprehension. Whole utterances in a mother’s baby-talk to very young infants tend to be short (about 0.5–0.75 s) (Malloch, 1999). They are typically repetitive and with rhythmic intonation and undulating pitch. The regular, simplified rhythms undoubtedly help the infant to synchronize (ibid.).

### Sensorimotor Integration, Overwhelm, and *Layering the Senses*

As we sensitively move on from *acclimatization*, beginning to add elements to our client’s rhythmic and sensory experience, we should do so only in small increments. We should add, remove, or adapt, just one layer at a time – *Layering the Senses* (Tortora, 2006). We need to be careful to observe which, and how much sensory input the client can tolerate and engage with. As functional integration occurs, we can extend sensorimotor experience in small-steps, layering in and out of the client’s thresholds of sensory vocabulary and tolerance.

### Summarizing Working Assumptions (2)

Piggy-backing on intact beat-perception pathways in autism, support for social timing should recruit tailored, information-rich rhythmic parameters to engage and clarify interactive pulse and relatable vitality. These lock-on parameters include beat specificity, periodicity, temporal and non-temporal accenting (including *relative acoustic startle*), and the use of *activation contours* (turbulent acoustic, and multimodal) as guides for perceptual-motor force-time curves. SMS support should facilitate good-enough synchrony as a basis for shared experience – enabling small-step co-regulation and a layered approach to sensorimotor integration. Initial and on-going environmental priorities should be acclimatization, simplicity, sameness, calm, short duration interaction, and rest. Interactive priorities should be: starting with the client’s spontaneous movement, sound, or object-play^20^; isolating, accentuating, and simplifying initial focus behaviors; scaffolding with rhythm; leveraging movement (including touch) and sound in combination to maximize rhythmicity; leveraging acoustic turbulence to encourage movement-response and to simplify processing load; then extending co-regulatory experience and sensorimotor integration through small increments within a varied tonescape.

We acknowledge the pervasive nature of timing and sensorimotor disturbance in autism. We are open to the possibility of rhythm-mediated SMS *entrainment* in interaction, yet expect that (as was found by Dvir et al., 2020) such support will take the form of temporary *scaffolding*. The aim here then, is to facilitate small-step co-regulation and sensorimotor integration within a zone of proximal synchrony.

## The Rhythmic Relating Skill Set

*Rhythmic Relating* offers a *skill set*^21^ that can be flexibly applied, as and when feels useful, when supporting playful interaction with a client with Autism^(Therapeutic Needs)^. The skill set can be used independently, or in support of the *play progression* we present in Section “The rhythmic relating play progression: building games together (from movement, sound, or object-play).” The benchmark here is the essential quality of co-creating, and passing through experiences together – synchrony for its own sake. There are no interactive expectations, fixed rules for progression, tick lists or programs. Rhythmic Relating is about facilitating realistically short moments of playful synchrony whilst respecting overwhelm, and the need for rest and withdrawal.

Phrases of playful interaction might be led by the client, sometimes the practitioner, often (and ideally) both in co-creation. In practice, the question of who is leading, is of far less significance than the sense of togetherness in synchrony. If the phrase is practitioner-led, then what is important is that the client is *actively experiencing* in relative synchrony. Or, if the phrase is client-led, then the practitioner should be *actively following*, ready to respond and develop. As practitioner, we can keep in mind this rule of thumb: *follow-lead-follow* (Hughes, 2004, 2011). We *follow* the client’s momentum, yet we feel free to take an initiating *lead* when it feels appropriate, then *follow* again when the client picks up the flow in response or lets us know that we have missed the mark – always careful not to coerce the client.

### An Overview of the Rhythmic Relating Skill Set

**FIGURE 3:**
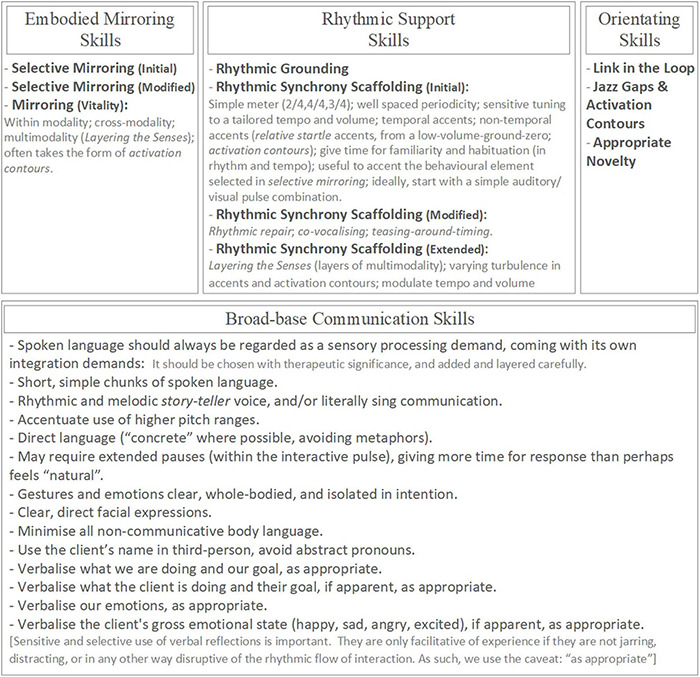
The Rhythmic Relating skill set: overview.

Please refer to the overview of the Rhythmic Relating skill set (Figure 3).

### Embodied Mirroring Skills

In what follows, we outline a series of reflective engagement skills which range from *Selective Mirroring (Initial)* (with close affiliation to simple imitation), through to a fully embodied, multimodal reflection of the client’s vitality in *Mirroring (Vitality)*. The range represents our small-step priorities: initial simplicity, low arousal, sameness, isolation and accentuation of one particular aspect of the client’s movement/sound/object-play; then playful development; with extension of co-regulation and sensorimotor integration through sensitively *layering the senses*.

In deciding which skill to use and when, it is important for the practitioner to act from a place of embodied observation, attempting to immerse themselves in how the client is experiencing and communicating. Isolated, simple mirroring may be perceived as a welcome simplicity, clarity, and validation to the client with Autism^(Therapeutic Needs)^. Or it risks being perceived as judgmental and patronizing. We need to spend time attuning to find what is helpful, and always remain sensitive to adjusting, based on felt-sense feedback from the client’s embodied communication.

#### Selective Mirroring (Initial)

We isolate a selected aspect of the client’s movement or sound to mirror. We might select a behavior, gestalt, or motif for its clear, repeating nature, or because it is marked by a noticeable emotion, mood tone, or level of arousal. We want to hone in on this behavior, bring awareness to it, accentuate its affective and expressive tone (we might exaggerate or diminish our reflection for emphasis), and minimize other elements of our behavior to allow this to stand out. Here we can use big distinct movements and/or clear amplified sounds as appropriate, later, progressing toward more nuance.

#### Selective Mirroring (Modified)

When developing a game, we will need to be able to modify our interactive responses without losing connection with the client. While matching the overall quality of the client’s behavior, we can play with modifying our mirrored movement or sound, *one aspect at a time*. Here are some aspects to consider. In movement^22^ : *effort* (pace, weight, fluidity); *mood* (emotional/energetic tone); *structure* (use of body as a whole vs. in parts; place of initiation of movement; placement/movement of limbs in relation to torso; upper-lower body relationship; left to right body relationship; contralateral body relationship; place of initiation of movement); *space* (proximity – near, mid, far reach; height level changes). In sound: volume (use of accents, crescendo vs. decrescendo); pitch (contours of sounds); timbre (breathy, soft, or vibrato voice or sound); form (musical motif or pattern); tempo (fast vs. slow and changes in between); articulation/length of sound (legato, staccato, tenuto) (Bruscia, 1987; Wigram, 2004; Geretsegger et al., 2015). While maintaining the overall style, we can also play with either exaggerating or diminishing a mirrored aspect of the client’s behavior.

#### Mirroring (Vitality)

We use our *kinesthetic empathy*, all our observation skills, to feel our best-approximation of the whole-body/whole-sound vitality-form being communicated by the client in any one moment, in series, or in a repeating rhythm (Tortora, 2006; Eberhard-Kaechele, 2012, 2019; Koch et al., 2015). Vitality-forms (including stillness and inaction); psychoacoustic dynamics (including silence); and/or extended patterns of vital quality, can be “mirrored” in various ways – the energy, shape or contour of vitality being re-communicated *in essence* (Stern et al., 1985; Wigram, 2004; Stern, 2010; Daniel, 2017, 2019). When we mirror vitality, we often use *activation contours*. We can match the energy and intensity of the client’s behavior without imitating or overinvesting in the particular emotional tone. This enables a safe, congruent way to connect, without fueling negative emotional patterns. Vitality can be mirrored as:

*- An alternate expression within the same modality*, for example: a client’s slow, rhythmic hand clench-and-release matched by a whole-body contract-and-open; a client’s anxious moan matched by mixed-pitch bubbling sounds (up-swish contour in pitch and volume, with a high level of turbulence, matching the energy contour but without replicating or fueling the anxiety); a client’s violent jump matched by large body movements flowing from the core (matching the energy release without turning up the anger).

*- A cross-modal expression*^23^ (matching with a different modality), for example: the arc of a client’s arm movement matched by a down-swoosh contour in pitch and volume; the pulse of a client’s vocalization matched by a foot tap; a client’s sad vocalization matched with a sensitive whole-body folding; a client throws a pillow energetically at the wall, the arm movement matched in build-up by a playful vocalization (up-swish in pitch and volume) and then accompanied in the throw by a matching “whoooosh” (burst in pitch and volume).

*- A multimodal expression*, for example: the client is energetically pushing a toy car back and forth, practitioner adds a swaying vocalization while swaying themself; the client is pouring sand from their hand into a sand-tray, practitioner adds a vocal activation contour (stretching down-swoosh in volume and timbre – full of granular turbulence) and strokes both hands down the client’s arm.

A sensitive mirroring progression (through *selective mirroring (initial*), and on to *same modality*, *cross-modality*, then *multimodality mirroring (vitality)*, with playful *selective mirroring (modified)* along the way) is an important dimension of *layering the senses*. In conjunction with the layered use of *rhythmic support skills*, we suggest this progression represents a useful small-step approach to facilitating sensorimotor integration in playful interaction with clients with Autism^(Therapeutic Needs)^.

### Rhythmic Support Skills

#### Rhythmic Grounding

Wigram (2004) described how, in rhythmic/tonal grounding, the practitioner keeps a steady beat (simple meters – 2/4, 4/4, or 3/4 recommended) as a stable “anchor” for the client’s expressions. This can be done by humming, singing, simple beatbox, repeating words, with a percussive “instrument” (drum, box, body, floor, soft shaker), or by playing a bass tone. In the early phase of interaction, this may provide a hyper-aroused client (for instance, anxious in a new situation) with a sense of rhythmical containment. Preferably, we will soon be able to pick up and match the pulse inherent in the client’s current movement or sound, and our *rhythmic grounding* will morph into the practice of *rhythmic synchrony scaffolding*. Or, if we cannot find this pulse, we continue on with the possibility that our rhythmic grounding will become integrated into the client’s behavior, adding pulse and regularizing heterogenous elements. The more complex the client’s behavior, the less likely we will find that initial pulse – or at least, a pulse we can readily connect with *via* a regular beat. In this case, especially with loops of object-play, we suggest rhythmic grounding can bring a useful sense of containment and momentum and be a rhythmic foundation for other Rhythmic Relating skills (e.g., *link in the loop* and *jazz gaps*, see later).

#### Rhythmic Synchrony Scaffolding (Initial)

Rhythmic Synchrony Scaffolding (Initial) – is the use of rhythm (in any modality or combination) to match, accent, cue, augment, and develop the client’s pulse in movement and sound.

In *rhythmic synchrony scaffolding* we prioritize picking up and accenting the client’s spontaneous pulse in movement, sound, or behavior patterns. We bring our expression into closer rhythmical alignment with that pulse. We make our behavior more obvious, describing intention with rhythmic accents, cues, and contours; describing what is to come, just-ahead-in-time. We may also bring clarity and energy to add to the existing pulse. Ideally, yet with sensitivity to the client’s sensory preferences^24^, we will start with a simple modality combination^25^ : *auditory* pulse (humming, singing, simple beatbox, using percussive “instruments” [drum, box, body, floor, soft shaker], or playing a melodic instrument with percussive emphasis) and *visual/movement* pulse (repetitive movement from the practitioner, defined in space and proximity). We need, always, to be aware of our impact on the client. We need to start simply (and stay simple for as long as needed), to match and be led by changes in the client’s interactive arousal levels, and not to push a sense of urgency or overwhelm with our added pulse. This is very much: *follow-scaffold*-*follow*.

We can choose to *accent* a repeating aspect of the client’s movement, sound, or object-play. We will use simple meters: 2/4, 4/4, or 3/4. We will choose a well-spaced periodicity for our accents. For instance, if a client is swaying left to right we could accent each sway (within a 3/4 meter); if a client is hand-flapping we could accent each fourth flap (within a 4/4 meter); if a client is repeating a spoken phrase we could accent the start, end, or a rhythmically significant mid-point of the phrase (within a 4/4 meter); if a client is walking around in a loop we could accent each second step (within a 2/4 meter); if a client is sliding on the floor, pushing a toy bear, we could accent each end-point of the slide (within a 4/4 meter).

In *selective mirroring* we hone in on, accentuate, and bring awareness to a particular aspect of the client’s movement or sound. In *rhythmic synchrony scaffolding* we could choose to *accent* that same aspect.

We can improve our accenting with:

*- Temporal guiding information – the up-beat of, for instance, the iambic pentameter (remember, da-Dum, da-Dum, da-Dum, da-Dum – with the “da-” upbeat as guide).*

*- Non-temporal guiding information – we can explore the use of *relative acoustic startle* for focal accents; tailoring our ground-zero-volume to each client’s needs (often below average conversational volume i.e., <60 dB), employing the playful “shock” factor of immediate variation in pitch, volume, timbre; using drum-like bass tones; the hiss-factor of a high-hat-like-tone; the surprise of a higher-pitched machine-like pulse (e.g. a laser gun sound effect); “magical” pulses like bell and triangle tings. Here we can also bring in sound effects (animal noises, cartoon character refrains (Homer Simpson’s “Doh!” as a perfect beat), machine and vehicle sounds, impressions) and focus words as accents.*

We can use *activation contours* as guides to direct the client toward on-beat timing and toward our accents. Perfect for turbulent *activation contours* are any sounds with lots of inherent movement: glides; crescendos (including “whhhooop”-like sounds); variations on playful hissing (“ssss,” “ssshhh,” blowing sounds); sounds with high levels of randomized internal movement (bubbling noises, raspberries, tongue wobbles); contoured spoken words (with movement in pitch, volume, timbre); contoured sound effects (cars *vrroooom*, animals *squark*, *woof*, *growl* – and if you can manage an elephant trumpet…!); and cartoon characters are literally designed for this, Scooby-doo’s “Jshiiiicks! for example.

Here are two examples. Firstly, using 4/4, with high-energy acoustic startle to accent the two and four: one and “POW” and three and “POW” and…, tiiiIIISSSSHHHH “POW,” the contour uses a turbulent up-swish pattern rising in pitch, volume, and adding timbre, all guiding toward the “POW” accent. Secondly, using 3/4 with playful, fairy-tale acoustic startle to accent the strong beat with a magical “TIIING”: TIIING te te, TIIING te te, TIIING… SSSHhhhh, TIIING, te, te, the contour uses turbulent down-swoosh, falling in pitch and volume, toward a whispery timbre, guiding intriguingly to the “TIIING” accent.

We can experiment with sticking with the same patterning for some time, leveraging support from familiarity and habituation.

#### Rhythmic Synchrony Scaffolding (Modified)

Once we have established a scaffolding pattern in which our focal accent is a *selected* aspect of the client’s behavior – for example, a vocalization - we could adjust the rhythm to bring our reflection of their vocalization into closer synchrony with our shared forward-moving pulse (and therefore, *complementarity*). This can function as *rhythmic repair* of rhythmic irregularity (Nielsen and Holck, 2020). This can also evolve into *co-vocalizing*, where we mirror elements of the client’s vocalization, bring it into a rhythm, and make up “songs” (verbal or non-verbal) as a development. We have found the practice of co-vocalizing to be a useful step toward turn-taking.

Our use of a *link in the loop* (see below) relies on establishing a shared rhythmic pulse (creating expectation and momentum) and then playfully “teasing” the client’s expectation. More generally, this type of *teasing-around-timing* is a crucial early developmental ingredient when building and developing games (e.g., peepbo” vs. “pee….p-bo!” vs. “pe-ee……..p-bo!”) (Wimpory, 1995, 2015).

#### Rhythmic Synchrony Scaffolding (Extended)

As the richness of client-practitioner interaction develops, we may want to add layers of multimodality to our scaffolding (*layering the senses*). In addition to *auditory* and *visual/movement* pulse, this could include *physical* pulse (varying type, pressure and position of contact and touch), or facilitating *proprioceptive* and/or *vestibular* pulses for the client (through assisted movement – possibly using supportive equipment such as blankets, trampolines etc.). In our use of *relative startle accents* and *activation contours* we can add additional layers of multimodality and vary the range and tone of turbulence. We can play with modulating the tempo and volume of our scaffolding.

### Orientating Skills

#### Link in the Loop

To promote an orientation, or if things feel stuck, one option is to add an element to the loop. The aim is to do something with relevance to the client’s movement, sound, or object-play, yet appropriately novel (the *right level of different* – noticeable, perhaps humorous, but similar enough not to jar). We repeat this simple action or sound with regularity, at the same point in a loop. If pitched and timed well, our action or sound can become part of the loop – an integrated link. We have become essential to the completion of the loop. Here are some examples:

*- With movement*, client is running a circuit of the room, touching various points on the wall in a loop, practitioner adds *rhythmic grounding* and positions themself with their hand directly over one of these touch-points.

*- With sound*, client is humming and swaying, practitioner adds *rhythmic synchrony scaffolding* and adds their own movement (an activation contour, quick-fade in embodiment, a whole-body withdrawing and shrinking small) every fourth bar of a 4/4 meter.

*- With object-play*, client is sliding across the floor on their bottom, pushing a toy bear with their feet, practitioner adds *rhythmic synchrony scaffolding* and then creates a human-bridge (arched on all fours) for the client to pass through in their current trajectory.

*- With movement*, client is rocking, practitioner adds *rhythmic synchrony scaffolding* and rocks in synchrony, then taps the client’s right hand on every fourth rock.

*- With object-play*, client is pushing a toy train around a track, practitioner adds *rhythmic grounding* and, each time the train passes the station, starts to chase the train with a toy car.

The link can replace an accent or phrase within *rhythmic grounding* or within *rhythmic synchrony scaffolding* (with or without a preceding *activation contour*). Alternatively, without rhythmic support, we can use a stand-alone *activation contour* in the lead up to a link. Then, from there, we can facilitate an orientation from the client, by either withholding the link (a *jazz gap* – see below); or changing our action or sound (*appropriate novelty*).

#### Jazz Gaps and Activation Contours

A *jazz gap* refers to a pregnant pause deliberately interjected into the rhythmic flow of communication. A jazz gap holds a silence longer than the natural on-beat demands. It has the energy of needing to be filled. A beat is expected, movement is compelled. We can play with the held duration of a jazz gap over a range up to around 6 s. If we push the duration much past this range, we lose the rhythmical impetus of the “here and now.” “The current consensus in music psychology and cognitive neuroscience is that the ability to associate beats, or perform them meaningfully as a pulse, stops at around 6 s or 0.16 Hz… It is at this point that the mind and body can no longer “lock on” – either actively through playing, or passively through listening – to the rhythm as a pulse” (Osborne, 2017, pp. 18–19). We have not found studies which could provide equivalence for individuals with autism. As such, we will proceed with the tentative working assumption that the 6 s window is appropriate for the high-end of a sense of associated rhythm. Practically, it will be essential for the *practitioner* to retain their sense of the continued rhythm, and so the 6-s high-estimate remains entirely relevant to the therapeutic tool.

We can use a jazz gap within *rhythmic grounding* or *synchrony scaffolding*. We simply replace an accent or short phrase. For example, in a straight 4/4 where the two and four are accented “TISH” sounds (employing *relative acoustic startle*): One and TISH and Three and TISH and One and [Jazz Gap…]. Or with the temporal accent of an iambic pentameter – da-Dum, da-Dum, da-Dum, da- [Jazz Gap…]. Or we can introduce a jazz gap within, or at the climax of an *activation contour*. For example, in a 3/4 swaying waltz: Dum, tuh, tuh, Click, tuh, tuh, Dum, tuh, tuh, Click, tuh, tuh, whooooaaahh…Click, tuh, tuh, Dum, tuh, tuh, Click, tuh, tuh, whooaaaah… [Jazz Gap]. We can also use a jazz gap to replace an established link in the loop (see above).

## The Rhythmic Relating Play Progression: Building Games Together (From Movement, Sound, Or Object-Play)

Rhythmic Relating is about free-flow playful interaction. The *play progression* we describe, is a template which can be used flexibly. The *progression* may be useful in extending spontaneity toward experiences which facilitate co-regulation and SI. It may also provide a helping hand when things feel stuck.

### An Overview of the Rhythmic Relating Play Progression (in Pictures)

**FIGURE 4:**
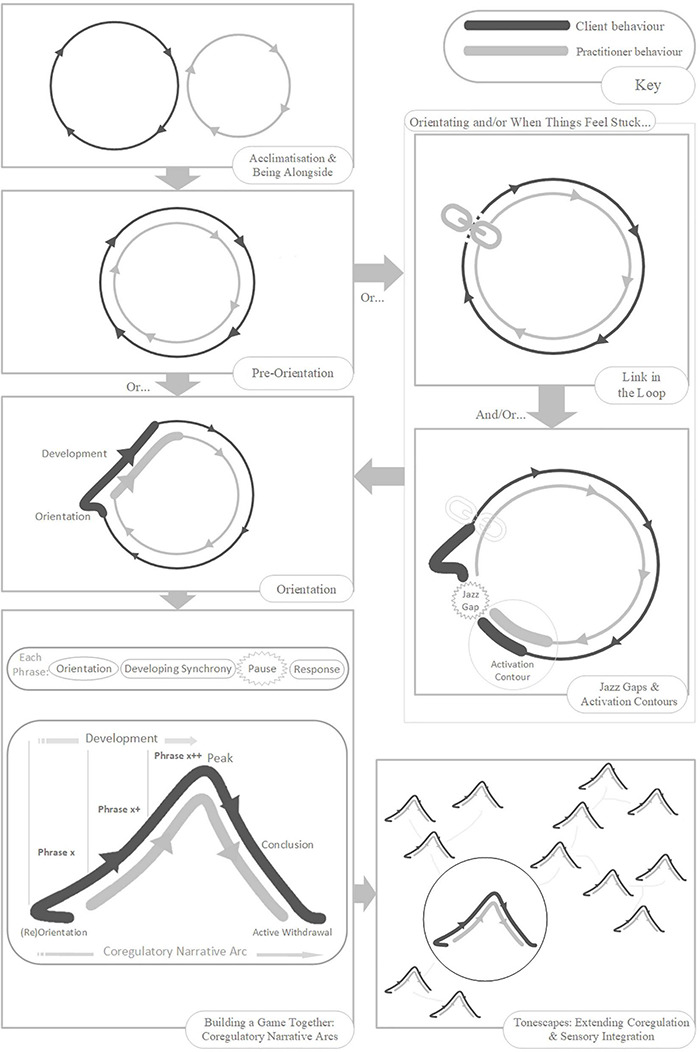
The Rhythmic Relating Play Progression: An overview (in pictures).

Please refer to the overview of the Rhythmic Relating Play Progression (in pictures) (Figure 4).

### An Overview of the Rhythmic Relating Play Progression (in Words)

**FIGURE 5:**
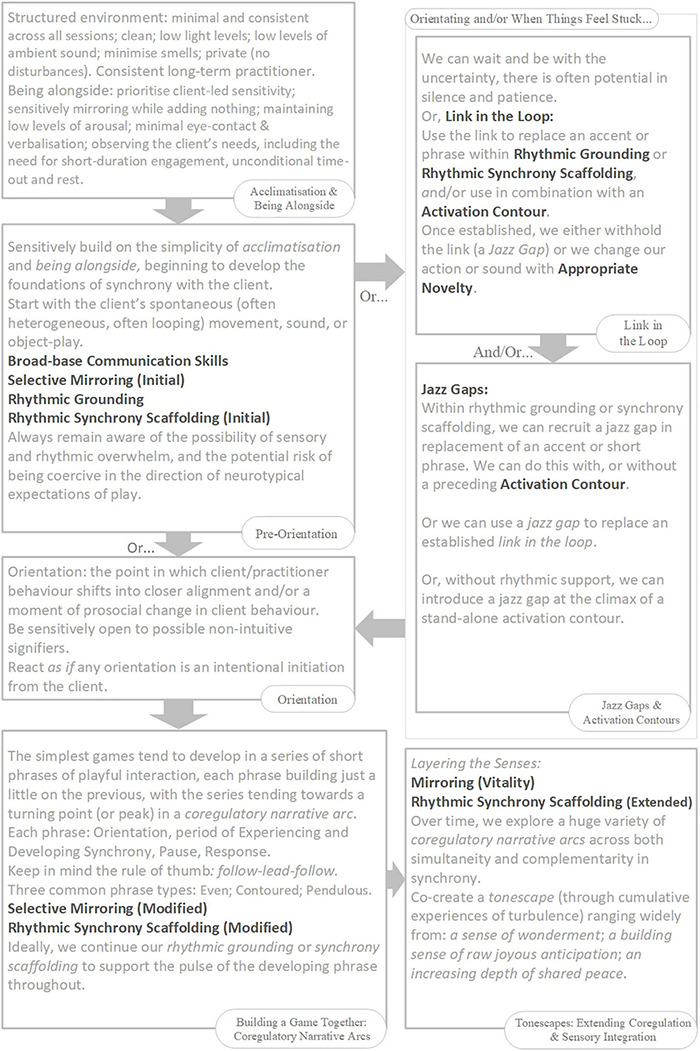
The Rhythmic Relating Play Progression: An overview (in words).

Please refer to the overview of the Rhythmic Relating Play Progression (in words) (Figure 5).

### Notes on the Rhythmic Relating Play Progression

#### Broad-Base Communication Skills

Applicable throughout – see Figure 3.

#### Acclimatization and Being Alongside

See section “Acclimatization, Simplicity and Sameness”.

#### Pre-orientation

We ask ourselves, “what might it feel like to be experiencing this moment through the client’s particular structuring of their movement, sound, stillness, and silence?” We aim to become sensitive to the pulse and quality of the client’s behaviors. We try to tune into energy, effort, momentum, emotional color, potentially non-intuitive time-frames for response, and direction of focus. We are attempting to attune to possibilities of shared meaning that we might not initially recognize or understand.

We start with the client’s spontaneous movement, sound, or object-play. We attempt to connect with a particular aspect of their behavior – s*elective mirroring (initial)* (section “Embodied Mirroring Skills” and Figure 3). If we are able to pick up on a repetitive, somewhat looping quality within the client’s movement, sound, or object-play – use *rhythmic synchrony scaffolding (initial)* (section “Rhythmic Support Skills” and Figure 3). If not, we can use *rhythmic grounding* to add pulse (section “Rhythmic Support Skills” and Figure 3). In our rhythmic support, we should be led by the client’s pulse and momentum where possible. If we are adding our own interpretation of pulse, we should be responsive to the possibility of rhythmic overwhelm (getting the momentum and mood wrong).

#### Orientating

If things feel stuck, we can wait and be with that uncertainty. There is often potential in space and patience. Or, as a possibility – use *link in the loop* (section “Orientating Skills” and Figure 5) and/or *jazz gaps and activation contours* (section “Orientating Skills” and Figure 5).

#### Orientation

**FIGURE 6:**
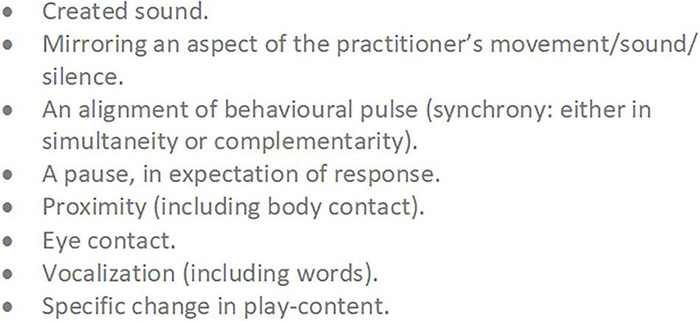
A non-exhaustive list of possible ways a client with Autism^(*TherapeuticNeeds*)^ might initiate an orientation.

In the Rhythmic Relating play progression, we have deliberately not defined a particular moment of initiation or specified an initiator. Instead, we are interested in the practitioner using the skill set, remaining sensitively open, and facilitating potential for bidirectional SMS. We consider the overt beginnings of an interactive phrase to be either, the point in which client/practitioner behavior shifts into closer alignment, or a moment of prosocial change in client behavior. Part of our practice is a continual tuning-in to possibly non-intuitive orientations from the client (Figure 6).

We also recommend the highly powerful practice of reacting *as if* any orientation is an intentional initiation from the client. This practice is a safety-net, ensuring we don’t miss opportunities, and also promotes positive feedback loops for learned interactive behavior.

#### Building a Game Together: Developing Co-regulatory Narrative Arcs

The simplest games tend to develop in a series of short phrases of playful interaction, each phrase building just a little on the previous, with the series tending toward a turning point (or *peak*) in a *co-regulatory narrative arc* (Figures 1, 4).

**FIGURE 7:**
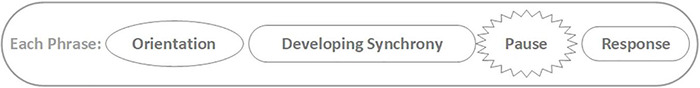
A *Phrase* of playful interaction.

Each phrase (Figure 7) starts with an orientation. Broadly, there are three possibilities at this point. The orientation itself could be a shift toward synchronous alignment, with the client’s behavioral pulse somewhat locking into the attempts of rhythmic facilitation made by the practitioner. Then there are two possibilities that come from the use of *activation contours* and *jazz gaps* (within our rhythmic supports): the client may have been compelled to move, to do something new; or, if we have introduced a *link in the loop*, the client may pause as something specific is expected of us.

As we are led into a shared period of developing synchrony, the type of orientation helps us respond and somewhat defines the terrain (the rough shape of the phrase to come: *even*, *contoured*, or *pendulous*):

- An *even phrase* is marked by both partners traveling alongside each other, through an even pulse, in simultaneity (exactly in-sync): walking; jumping; rocking; blinking; sharing a regular sonic pulse; placing Lego bricks with regularity etc. After orientation and an extended moment of experiencing our even synchrony, we can pause and seek response (preferably), or respond ourselves through *selective mirroring (modified)* (section “Embodied Mirroring Skills” and Figure 3) and or *rhythmic synchrony scaffolding (modified)* (section “Rhythmic Support Skills” and Figure 3). Then, a further orientation, and the next iteration of the phrase.

- A *contoured phrase* is one defined by an *activation contour*. Again, this experience is defined by simultaneity and traveling through the contour together. Either partner may be responsible for generating the activation contour, the other might be engaged in a relatively receptive fashion. What is important is that there *is* engagement and a sense of shared experience. After an extended moment of experiencing our contour together, we can pause for response. Contoured phrases tend to develop through increments of intensity and anticipation, tend to be repetitious in nature, building toward an overall crescendo peak (see section “What Does Disrupted Bidirectional Social-Motor-Synchrony Look Like in Play?”). The response, therefore, tends to be a subtle variation of “AGAIN”! However, there is also impact in occasionally breaking the expected rhythmic build-up through *selective mirroring (modified)* and/or *rhythmic synchrony scaffolding (modified)*.

- A *pendulous phrase* is one specifically defined by action, then response, in complementary synchrony within a shared time-frame – the simplest being the back and forth of turn-taking, the more complex being patterns of response delayed or staggered in time. We use a variation of imitation in movement or sound – *selective mirroring (initial)* - or a repetitious action in object-play (throwing a ball; sliding a soft toy; pressing a switch etc.) to develop a turn-taking dynamic. After an extended moment in this shared synchrony, we pause for response and the next phrase. We can develop the game with *selective mirroring (modified)* and/or *rhythmic synchrony scaffolding (modified)*.

#### Tonescapes: Extending Co-regulation and Sensory Integration

We can facilitate sensorimotor integration, depth of relatable vitality, and a range of emotional and arousal experiences, all through our sensitive practice of *layering the senses* – *mirroring (vitality)* (section “Embodied Mirroring Skills” and Figure 3) and *rhythmic synchrony scaffolding (extended)* (section “Rhythmic Support Skills” and Figure 3). Over time, we explore a huge variety of *co-regulatory narrative arcs* (Figures 1, 4) across both simultaneity and complementarity in synchrony. Client and practitioner travel together in and out of the client’s thresholds of emotional vocabulary, arousal, and tolerance. These co-regulatory arcs are like hills and mountains in the *tonescape* we travel through together (section “Recruiting Acoustic Brain-Stem Turbulence: Evolutionary Sounds That Move Us” and Figure 4). We co-create this tonescape, through cumulative experiences of turbulence, ranging widely from:

*- A sense of wonderment* – light-footed variation in levels of turbulence in subtle playful combinations. Alternating between different types of activation contours. Moving across varying dimensions of quality. Finding a winding path to a turning point (peak) which could feel like a whispered moment of shared significance.

*- A building sense of raw joyous anticipation* – high levels of turbulence. Sticking with the same phrase with similar activation contours, building the level of arousal incrementally. Moving forwards with increasing anticipation and momentum to a high-energy peak in release and usually laughter.

*- An increasing depth of shared peace* – minimal yet playful turbulence. Just gently together. Moving toward a peak and extended conclusion in poignant silence.

### The Rhythmic Relating Play Progression: Examples in Practice

**FIGURE 8:**
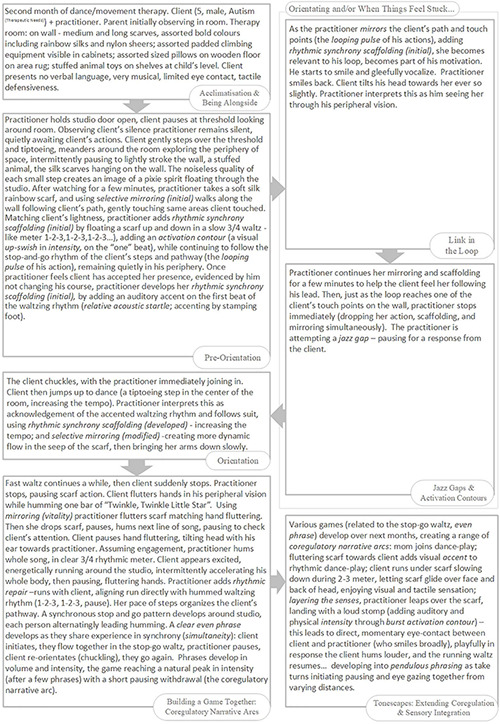
Case example. Rhythmic relating within dance movement therapy: Suzi Tortora.

**FIGURE 9:**
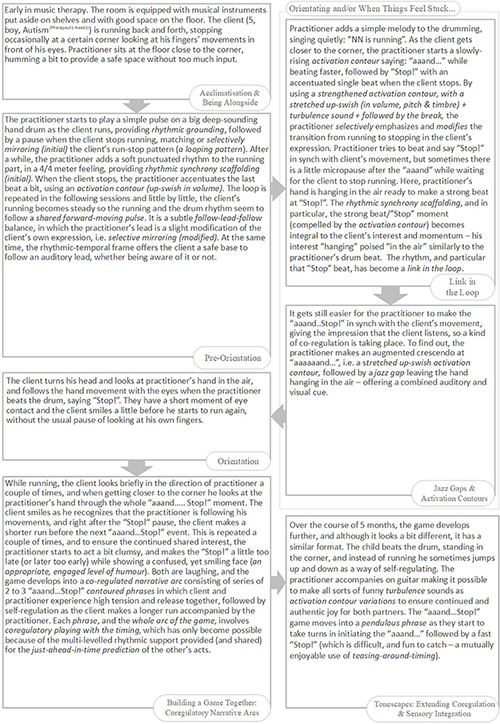
Case example. Rhythmic relating within improvisational music therapy: Ulla Holck and Monika Geretsegger.

**FIGURE 10:**
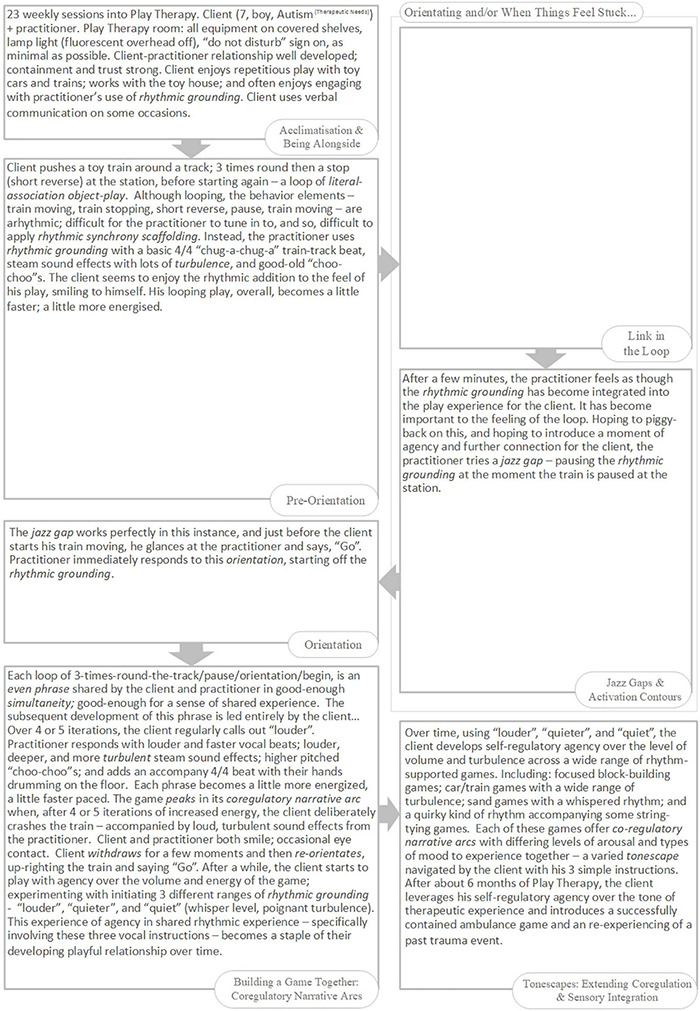
Case example. Rhythmic relating within play therapy: Stuart Daniel.

**FIGURE 11:**
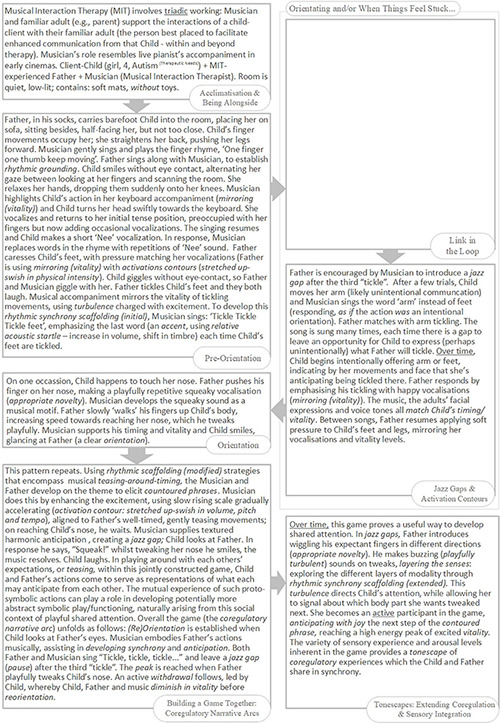
Case example. Rhythmic relating within musical interaction therapy: Judit Elias-Masiques, Marie-Claire Howorth, and Dawn Wimpory.

Please see the Examples in Practice (Figures 8–11) and Supplementary Material (Examples of Rhythmic Relating in Practice).

## Conclusion

We have reviewed evidence of disruptions to social timing, sensorimotor timing and integration in autism. We have discussed how such disruptions affect shared timing in play and intersubjective meaning-making, so important for learning, development, and health.

We have proposed *Rhythmic Relating*: a system which aims to augment bidirectional communication and facilitate good-enough social timing; opening up the possibility of playful therapeutic interaction, small-step co-regulation, and layered sensorimotor integration. We have designed Figures 3, 4, 5 (above) to be used together to provide a take-home working summary of the main features of the model. Through integrating *Rhythmic Relating* principles into a broadly child-centered therapeutic approach to interaction with clients with Autism^(Therapeutic Needs)^, we hypothesize that tailored rhythm-supported experiences of social timing will enable shared-meaning making, ease, and joy. Importantly, over time, we predict increased co- and self-regulation, reduced anxiety and challenging behavior, and greater trust in social relations.

We suggest a suite of pilot intervention studies is now needed to assess the possibility of combining *Rhythmic Relating* with different therapeutic approaches in playful work with individuals with Autism^(Therapeutic Needs)^. Such studies would assess therapeutic efficacy and allow for a fine-tuning of the model in real-world experience.

In addition, we propose two specific empirical hypotheses designed to clarify the significance of certain key features of the *Rhythmic Relating* approach:

(1) *Is rhythm a modulating factor of neural processing speed in autism?* Testing the impact of contextual ASR presentation (defined by rhythm; rhythm with activation contours as cues; and activation contours alone), on ASR magnitude and latency, in comparison to isolated startle stimuli (all variables presented at various volumes) – for subjects: HFA, TD controls.

(2) *Can acoustic Tau-G guide synchrony in autism?* Testing the impact of acoustic activation contours as Tau-G guides across various movement acts; each which define and test different potential synchronization dynamics - for subjects: HFA; TD controls.

Further, we propose that more research is required to better understand the underpinning neurobiology and neuromotor psychology disrupted in autism, *vis-à-vis* basic human intersubjectivity predicated on shared timing, feeling, and intention.

*Rhythmic Relating* aims to open up the therapeutic possibilities of play. We recognize such therapeutic experience as reflecting the intersubjective characteristics of typical preverbal interactions, with a conscious emphasis on shared, affective, and embodied experiences that may otherwise remain inaccessible for people with Autism^(Therapeutic Needs)^. The emotional and developmental significance of such opportunities should not be underestimated. The dance of interactive synchrony is, “the basis of social connection and empathy; it makes people trust and like each other” (Feldman Barrett, 2017, p. 287).

## Data Availability Statement

The original contributions presented in the study are included in the article/Supplementary Material, further inquiries can be directed to the corresponding author/s.

## Author Contributions

SD contributed as lead author, developed the initial premise and working concepts for the model and manuscript, at each stage worked with the co-authors to develop the model and manuscript, and contributed to the final edit and clinical examples of the model in practice. DW contributed as primary author and editor, developed the model, and contributed clinical examples of the model in practice. JD-B contributed as primary author and editor. SM contributed as editor and developed the model. UH, MG, ST, SK, JE-M, M-CH, and KS developed the model and contributed clinical examples of the model in practice. NO, BS, PD, MR, RS, KF, and PA developed the model. All authors contributed to the article and approved the submitted version.

## Conflict of Interest

The authors declare that the research was conducted in the absence of any commercial or financial relationships that could be construed as a potential conflict of interest.

## Publisher’s Note

All claims expressed in this article are solely those of the authors and do not necessarily represent those of their affiliated organizations, or those of the publisher, the editors and the reviewers. Any product that may be evaluated in this article, or claim that may be made by its manufacturer, is not guaranteed or endorsed by the publisher.

## Footnotes

1 Written in dialogue with Penelope ‘Pum’ Dunbar. Pum is an artist and creative researcher/thinker with autism (Delafield-Butt et al., 2021a,b).

2 Here we are suggesting that the primary route to self-regulation, especially for infants, is through physical and social co-regulation with another person. There are other routes, for instance: co-regulation with a calming animal; sensitive physical containment; taught techniques (such as breath work); rhythmical physicality etc. All of which may serve as powerful potential supports for individuals with autism.

3 Arousal, *the state of behavioral or physiologic activation*. https://medical-dictionary.thefreedictionary.com/arousal - accessed 10/11/2021. We note that the term *arousal* can have either negative or positive connotations. When used throughout this text, we will clarify with the terms: *Social Engagement System* arousal (positive) or *Mobilization System (Fight/Flight)* arousal (defensive/negative).

4 *Autistic Disorder/Childhood/Infantile Autism* (ICD-10 6A02: World Health Organization, 1993); *Autistic Disorder now included within ASD (ICD-11 6A02:* World Health Organization, 2019*).*

5 Our clarifying term, Autism^(Therapeutic Needs)^, relates closely to the DSM V (American Psychiatric Association, 2013) qualifier: level 3, i.e., “requiring very substantial support.”

6 We use the term ‘practitioner’ to include anyone working with the Rhythmic Relating approach.

7 We use the age-non-specific term “client,” to refer to anyone for whom Rhythmic Relating is developmentally and therapeutically appropriate.

8 The *vitality affect* of movement refers to an integrated perceptual, affective and expressive experience: the felt experience of an intentional flow of action combining movement, force, and direction altogether with intention and feeling (Stern, 1999, 2010).

9 Throughout this paper, a citation date with a superscript * (e.g., Jones, 2019*) denotes any autism-specific study or meta-analysis in which cognitive level(s) are unspecified and/or where LD may be a confounding variable. Such studies, and their inclusion with an asterisk here, reflect changing emphases in research priorities over time. Autism-specific studies which are controlled for LD and/or other neurodevelopmental concerns (for instance, though the inclusion of a cognitively matched control group, the removal of certain subjects from the design, or through employing, observing, or discussing only subjects with High Functioning Autism), we leave without a superscript. Autism-related hypothesis/theory articles and non-autism specific studies and articles, we also leave without a superscript.

10 *Vitality-form* is synonymous with *vitality affect* (Køppe et al., 2008; Stern, 2010).

11 A note on interpreting functional imaging (fMRI and fNIRS) data: Isolated interpretations of the relationship *between* activation level (hyper or hypo) in any one brain region *and* functionality of that region are extremely limited. For instance, hyperactivation of the IPL in autism could equate to: the IPL working well; the IPL working hard to compensate for internal or external dysfunction; or the IPL being disrupted by too much internal neural activity (i.e., overproduction in which functional connections are lost, Courchesne et al., 2001, 2003). To gain a clearer picture, we triangulate: TD/HFA comparison of IPL function; TD/HFA IPL-related behavioral comparisons; and any (dys)functional overlap between the various functional networks which recruit the IPL.

12 Alterations of insula gray matter volume have been reported in individuals with ASD, as compared with TD individuals (Kosaka et al., 2010; Cauda et al., 2011; Ecker et al., 2012).

13 Diagnosed at 3 years old, *Infantile Autism* (ICD-10 6A02: World Health Organization, 1993); later clarified, HFA - author’s confirmation, 2022.

14 Corresponding to the extended “now”, or the *psychological present moment* (Stern, 2004).

15 Our categories here, are closely akin to Feldman’s *matching synchrony* and *sequential synchrony* (Feldman, 2007a,b). Our categories have been further informed by detailed classifications within Dance/Movement Therapy (Behrends et al., 2012; Eberhard-Kaechele, 2019) and Improvisational Music Therapy (Bruscia, 1987; Wigram, 2004).

16 Clarifying terms from, https://dictionary.onmusic.org: • Rhythm: “The controlled movement of music in time. It may be defined as the division of music into regular metric portions; the regular pulsation of music.” • Beat: “The regular pulse of music which may be dictated by the rise or fall of the hand or baton of the conductor, by a metronome, or by the accents in music.” • Meter: “Measure of time; the grouping of beats into regular patterns.” Accent: “The principle of regularly recurring stresses which serve to give rhythm to music.”

17 For this specific purpose we rule out Kaur et al., 2018, as their synchronization tasks were all complex multi and bilateral coordination tasks.

18 Participants with ASD were slower to gain the benefits of habituation, needing a longer period before ASR latency effects were minimized, as compared with age-matched TD controls (Ornitz et al., 1993*).

19 Our categories are synthesized and developed from Stern (1993; 1999; 2010).

20 Children with autism rarely display “symbolic” play (the imaginative use of one unrelated object to represent another). They do engage in two types of object-play: simple physical manipulation; and/or realistic (literal-association) play. The latter is contextually relevant play with a toy that has recognized identity: e.g., playing with a toy train as train (with sound effects, train behavior etc.). We combine both types within the term, *object-play*.

21 The presented *skill set* is based on the preceding and subsequently cited research and the combined therapeutic and research experience of the authors.

22 *Rhythmic Relating* draws on ideas from *Laban Movement Analysis* (LMA or L/BMA) (Laban, 1976; Bartenieff and Lewis, 1980); Tortora’s related *Ways of Seeing* model (Tortora, 2006); and Eberhard-Kaechele’s developmental mirroring taxonomy (Eberhard-Kaechele, 2012) in considering the key qualitative, non-verbal elements of the client’s movements. We have minimized and simplified related categories to fit the *Rhythmic Relating* priorities, and to support practitioners not trained in specific movement approaches.

23 Akin to Stern’s *attunement* (Stern et al., 1985; Stern, 2010).

24 Remember *pulse is amodal* and can be communicated *via* any sense. Adapt as is needed for your client.

25 Here we describe the senses from the client’s perspective.
