# Supplementary material for: Rhythmic Relating: Bidirectional Support for Social Timing in Autism Therapies
Source: Front Psychol. 2022 May 16;13:793258. doi: 10.3389/fpsyg.2022.793258 (PMC9186469; doi:10.3389/fpsyg.2022.793258)
Supplement: Supplementary file 1 [file Data_Sheet_1.PDF]

8 weekly sessions into Play Therapy. Client (4, boy, Autism (Therapeutic Needs)) + practitioner. Play Therapy room: all equipment on covered shelves, lamp light (fluorescent overhead off), "do not disturb" sign on, as minimal as possible. Client has been engaged in energetic exploration and manipulation of the room for several sessions – minimal contact with practitioner. Client is sliding across the floor on his bottom, sliding a pillow forward with both feet – a short, repetitious behavior loop.

Acclimatisation & Being Alongside

Practitioner spends some time copying the client's physical action, *selective mirroring (initial)*, sliding on the floor alongside. No observable response from the client. The client's loop holds a behavioural pulse (loose, but rhythmic enough to follow and predict), a pattern of slide-beat-slide-beat-slide…

Feet Slide (pillow forward)	Slide Stops	Client Slides (closes gap to feet)	Slide Stops
Stretched Slide	Beat	Stretched Slide	Beat

Practitioner continues *mirroring* and adds *rhythmic synchrony scaffolding* (quiet, simple beat-box)…

Feet Slide (pillow forward)	Slide Stops	Client Slides (closes gap to feet)	Slide Stops
Stretched Slide	Beat	Stretched Slide	Beat
Practitioner. Vocal Activation Contour: "Tisssss…	Vocal Beat: …KA"	Practitioner. Vocal Activation Contour: "Whooo…	Vocal Beat: …PA"

No observable response from the client.

Pre-Orientation

Orientating and/or When Things Feel Stuck...

On the basis of *follow-lead-follow*, practitioner tries a playful experiment. Practitioner attempts a *link in the loop*: just before the client slides the pillow forward with his feet, practitioner kneels sideways on (to the direction of motion) and makes an archway with their back and arms, an arch for the pillow to slide through. Client pauses, and then continues his loop – now sliding the pillow, and then himself, through the human archway. In response to the contained feeling of the archway, practitioner now continues their *rhythmic synchrony scaffolding* at a whisper, changing the beats to higher-pitch, lower volume "piiings":

Feet Slide (pillow forward)	Slide Stops	Client Slides (closes gap to feet)	Slide Stops
Stretched Slide	Beat	Stretched Slide	Beat
Practitioner. Vocal Activation Contour: "shhhh…	Vocal Beat: …piing"	Practitioner. Vocal Activation Contour: "shhhh…	Vocal Beat: …piing"

Link in the Loop

The client seems engaged with the new archway-loop, has now forgotten the pillow and is enjoying sliding through the human archway as a playful action for its own sake. Client and practitioner do this a few times. Then, in an attempt to give the client agency and to encourage an orientation, the practitioner introduces a *jazz gap*. They hold back from making the archway, waiting for response.

Jazz Gaps & Activation Contours

The client looks at the practitioner, making direct eye contact for the first time. Practitioner immediately responds, makes the human archway, continues their whispered *rhythmic synchrony scaffolding*, and the loop continues.

Orientation

Client and practitioner share this *contoured phrase*, in *simultaneity*: Client experiences an *activation contour* (a *stretch in embodiment* and, relative to his movement through the archway, an *up-swish in proximity* - a *visual looming experience*), directly in synchrony with the practitioner's *vocal activation contour* (the whispered "shhhhh…", a *stretch in pitch and timbre*). Next, the practitioner adds something. With each iteration of the *play phrase* the practitioner shrinks the human archway a little smaller (adding to the experience of *visual looming* and *containment* for the client), and adapts their *vocal activation contour* accordingly - from a whispered *stretch* to a vocal *down-swoosh*, a loud whisper tailoring away down to silence:

Feet Slide Forward (under arch)	Slide Stops	Client Slides Forward (under arch)	Slide Stops		Eye Contact
Stretched Slide	Beat	Stretched Slide	Beat	Jazz Gap	
Practitioner: Vocal Activation Contour: "shhhhh…	Vocal Beat: …piing"	Practitioner. Vocal Activation Contour: "SSSShhhhhh… + Shrinks arch	Silent Beat: …..”	Jazz Gap	Practitioner: Remakes arch

Building a Game Together: Coregulatory Narrative Arcs

The game builds over several iterations of the *phrase*; each initiated by the client's direct eye contact; each defined by a smaller human archway, until the client is wriggling through a small tunnel, to break out triumphantly on the other side. This is the *peak* of the game (client and practitioner laughing together) and is followed by a short period of self-regulatory *withdrawal* by the client, and then a quick re-orientation (eye contact) and the next game.

Tonescapes: Extending Coregulation & Sensory Integration

Supplementary Figure 1. Case Example. Rhythmic Relating within Play Therapy: Stuart Daniel.

Seven weekly sessions into music therapy. Room equipped with musical instruments on shelves, with good space on the floor. As the client (4, boy, Autism [Therapeutic Needs]) enters the room, he brings a stack of square picture cards with him. His attention is focused on the cards as he puts them down on what happens to be a conga drum. Practitioner sits on floor, observes, hums lightly to provide a calm, relaxed atmosphere (*rhythmic grounding*).

Acclimatisation & Being Alongside

The client continues to be focused on the cards, taking single cards from the stack in his hand, looking at them, and then putting them down or letting them fall on the floor, without any apparent intention to use the cards in an interactive/communicative way (a short, repetitious *loop* following a *loose pulse*). The practitioner stops humming, moves to the conga drum next to the client's one, and starts to play in a slow 2/4 meter that accompanies the client's movements, i.e. *rhythmic synchrony scaffolding (initial)*: When he picks up a card, the practitioner plays 'Dum-dada-dum' (like a short question with a sense of asking "where should this go?"), and if the client puts the card down on the drum skin or lets it fall, the practitioner follows up with a response-like 'Dadadada-dum' (as if stating "now this one is there"). This *activation contour* (the drum-beat 'comments', an *up-swish in intensity*, followed by a *down-swoosh in intensity*) uses *selective mirroring (vitality)* – matching the contoured vitality of the client's movements with the cards.

Pre-Orientation

Watching the practitioner play, client starts jumping up and down with increasing engagement and enjoyment and in eager anticipation of the cards not just jumping up and down, but falling off. As they finally do, he laughs out loud in excitement again. To make the practitioner continue, client takes practitioner's hand and moves it to his drum (a clear *orientation*).

Orientation

Client keeps jumping up and down while practitioner beats the drum using crescendos and the *jazz gap* to wait for the client's reaction; client excitedly exclaims "louder!", practitioner follows this by beating the drum so that the cards fall off. After a few rounds, all the cards are gone from the drum skin, practitioner makes a "what now?" gesture. Client finds a new card and puts it on the drum. Practitioner tries *modification* and offers the client a turn to play. He walks around for a moment, then back to conga, starts hitting it with both hands until the card falls off. He laughs briefly. Practitioner asks for a new card, and as the client has placed it on the drum, waits for him to have another turn. However, the client says "you!", and walks around some more. Practitioner starts playing (an example of *follow-lead-follow*), and the client commands "louder!". Practitioner plays a few beats so that the card jumps once. Client joins in jumping, requesting "very loud!". Practitioner plays strong beats, then stops again. Client exclaims "even louder!" – finally the cards fall off, which is met with delight by the client.

Building a Game Together: Coregulatory Narrative Arcs

Orientating and/or When Things Feel Stuck...

Practitioner starts to modulate the volume of drum playing, adding a little crescendo in every first part, followed by a more quiet/decrescendo second part (each crescendo/decrescendo being an up-swish or down-swoosh *activation contour* respectively) – rhythmic *synchrony scaffolding (modified)*. Client looks up from his cards, and in response to this increase in awareness, the practitioner attempts a *link in the loop* by playing the last, strong beat of the first, 'questioning' rhythmic motif on the client's drum. Caused by this, the few cards lying on the drum are flung up in the air before falling down on the drum skin again. The client notices this, and as the practitioner plays the motif again with a stronger crescendo, some of the cards fall on the floor. Client and practitioner laugh out loud.

Link in the Loop

The practitioner puts a few cards back on the client's drum and continues to play, starting on the practitioner's drum, with the last beat of the first part on the client's drum, then retreating to the own drum again. The first few times, the beat on the client's drum is too weak to make the cards fall off the drum. Gently teasing, the practitioner extends the first part to increase the build-up of the arc of suspense (Dum-dada-dadadada-dadadada-dadadada-Dum!) and after a few more times also adds a *jazz gap* before the decisive beat, keeping the hand in the air, which further increases the suspense.

Jazz Gaps & Activation Contours

The game comprises a series of *phrases*: *Orientation* is followed by the practitioner's drumming and the client's excited jumping *contouring in intensity together* (a shared build-up) – practitioner using *selective mirroring (vitality)* – each *peak* (all cards off the drum, joint laughing) followed by a *pause*, and a *proactive withdrawal*: Both teasingly negotiate who is going to make the next move (drum beat / jump) = *orientation* to start the next game. Thus, each game provides a *co-regulatory arc* with varying levels of arousal, modulated by more or less dramatic tension (volume, tempo, *jazz gaps*). Adding *multimodality*, practitioner also uses vocalization, with the vocal sounds matching the respective *activation contour* – *mirroring (vitality)*.

Tonescapes: Extending Coregulation & Sensory Integration

Supplementary Figure 2. Case Example. Rhythmic Relating within Improvisational Music Therapy: Monika Geretsegger & Ulla Holck.

Third month of Dance Movement Therapy (DMT). Client (5, female, Autism (Therapeutic Needs)) + practitioner. Client tolerates touch. Therapy room: soft floor mats – a big donut, triangular shaped soft element, two gymnastic mats, a big mirror at adult belly height; an open cupboard with toys, balls, bean bags, animal toys. Client presents no verbal language, limited eye contact, likes to repeatedly sing "Twinkle, twinkle, little star", highly stereotypic, no progress in the first three months.

Acclimatisation & Being Alongside

Client enters the room, and first meticulously needs to re-organize the room to the way she knows it, pulls mats to the one side of the room next to each other, puts triangular soft element at its place, shifts donut. Then walks around the room in circles, using the mats and triangular element. No interaction. Practitioner initially observes client's actions. Client steps on the soft elements in the *same pattern* for three rounds (a behavior *loop*). The repetitive quality leaves an image of an obsessiveness with order in the studio. After watching for a few minutes, practitioner decides to accompany the child's movements with vocalizations filled with matched intensity and rhythmic *quality – cross-modal mirroring (vitality)*, such as an accelerating "run, run, run, run, run". And, as the child stops, "stop, pause". Which becomes a pattern, with the "run, run, run, run, run's" as a regular auditory mirroring, matching the client's movement quality, and the stop, pause with a down-beat, matching the client's drop of tension in these moments.

Pre-Orientation

Orientating and/or When Things Feel Stuck...

The above (Pre-orientation) behavior accompaniment, doesn't lead to enhanced interaction with client; client does not seem to notice. Trying something novel, with heightened sensitivity to *follow-lead-follow,* practitioner gently picks up client - attempting an altered, gentler, containing rhythm (always ready to adapt if this was not received as appropriate): rhythmically moving the client: up-drop, up-drop, up-drop, and pausing, in a clearer, more accentuated pattern than before. Their new shared positioning means that client and practitioner are now facing an actual mirror. The practitioner introduces *rhythmic synchrony scaffolding (initial)*, using vocalizations, aligning a "hop" with each physical up-drop; three "hops" then a pause. The hop, hop, hop is delivered with a staccato pulse, increasing in tension, the pause in a soft relaxed way, with decreased tension. After this additional tactile communication, and clearer accentuation of rhythm, child becomes more alert, ready to join, evidenced in matching tension changes in her body, first in her neck. The change in dramatic tension inherent in the "hop, hop, hop" build-up, and, in particular the moment of pause for release, becomes a *loop* engaging client – the pause, a particular *link in the loop*.

Link in the Loop

Practitioner continues for a few minutes to help client feel and integrate the rhythmic pattern. Breathing patterns between client and practitioner start to match. Client turns her head toward practitioner, gaze remaining downward. To create a jazz gap, practitioner pauses, after the second "hop" in a series, to see how the client might react.

Jazz Gaps & Activation Contours

The client holds her breath and looks briefly at practitioner with increased interest. A clear *orientation*, the practitioner interprets this as an action in service of a dialogue. Practitioner repeats the action of the "hop, hop" –void-, client gazes again with enhanced affect.

Orientation

Using *rhythmic synchrony scaffolding (modified)*, the practitioner softly sings to client, "hop, hop, shhhh"; both turn to the mirror; child's body relaxes into practitioner's arms. Continuing to hold her, practitioner uses second and third finger of her right hand to tap client's (left) arm, moving upward to child's shoulder during "hop, hop" and downward to child's elbow with "shhhh" using both fingers (here, using *mirroring (vitality)* and creating cross-modal *activation contours* – a *stretched up-swish* in *embodiment* and *intensity* as the taps move upwards accompanying "hop, hop"; a *stretched down-swoosh* as they move down with the "shhh". Intermittently, practitioner doubles the "hop, hop"s to "hopple, hopple"s, with two finger taps each (appropriate *novelty and modification*). Child stays alert with enhanced positive affect to this new *contoured phrase*. Practitioner maintains steady predictable beat. A feeling of increased togetherness arises. Practitioner continues with series of similar *contoured phrases*, each time re-initiating a *jazz gap* or the "shhh" – the game culminates in a "wiggle, wiggle, wiggle……", with hands of child and practitioner moving around one another. The heightened positive affect in both creates a *peak* to the interaction – fulfilling the *coregulatory narrative arc*.

Building a Game Together: Coregulatory Narrative Arcs

The basic, client-led sequencing of each session remains the same over 4-month treatment. In each session the client starts with ritualization (arranging the room, run, run, run, run, run … highly stereotypical behavior), feeling of being stuck continues with this section. Then follows a range of games – with a variety of *coregulatory narrative arcs*; a *tonescape* of shared arousal experience. In this main part of the sessions: practitioner develops *rhythmic synchrony scaffolding (modified) by* picking client up, holding and bouncing her, while singing and pointing to YOU and ME (multimodal *accents*); *layering the senses* (tactile – visual- auditory); looking for "me" and "her" in the mirror; and *selectively mirroring (initial)* her motor cues of approach/ avoidance. Overtime client relaxes more (softening muscle tone), sometimes shortens opening rituals, becoming interactive earlier. Singing supports co-regulation, creating interactional peaks, used as main interactive entry point until end of treatment.

Tonescapes: Extending Coregulation & Sensory Integration

Supplementary Figure 3. Case Example. Rhythmic Relating within Dance Movement Therapy: Sabine Koch.

Child-Client (3.5 yrs, boy with Autism (Therapeutic Needs)) + Mother (experienced in MIT) + Musician (Musical Interaction Therapist). Low-lit room: cupboard, sofa, mattress, *without* toys. Child follows brick lines on internal walls, with fingers sliding along them, walking at irregular pace. Child's eyes track his fingers while Musician and Mother vocalize 'swish swi-i-ish' to Child's rhythms with *selective mirroring (initial)*. Keyboard accompaniment supports *rhythmic synchrony grounding,* to contain Child's irregular walking pace.

Acclimatisation & Being Alongside

This behavior *loop* is established through many repetitions, *rhythmic synchrony grounding* continues. Mother matches Child's more regular walking pace, supporting Child's sensory exploration of wall (*selective mirroring (initial))*. Mother accentuates Child's finger movements by adding *activation contours* (*Up-swish & Stretched up-swish in pitch*) and Musician embeds her vocalized swishes in pitched contours on the keyboard *(selective mirroring (modified))*. Mother moves closer to Child, until her finger movements follow his, a few centimeters away. Child's movements, echoed by Musician's input, provide a good-enough flow for a pulse which is contained within the keyboard bass as *rhythmic synchrony scaffolding (initial)*. Child smiles to himself, appearing to enjoy the sensory nature of this experience. Familiarity to this routine becomes established. Musician layers musical accompaniment by decorating Mother's short 'swish' with rhythmic chordal *accents* and sustaining *pitch contours*, changing the timbre on Mother's longer 'swi-i-i-ish'. This enhances the overall sensory experience - *rhythmic synchrony scaffolding (extended*). Mother's vocal response becomes more confident and animated as it is transported by the music. There is dynamic flow in her body movements as she joins her child to share in his sensory experience of the wall. She gently attempts some swishes of her hand through Child's hair while continuing to swish the wall.

Pre-Orientation

Orientating and/or When Things Feel Stuck...

Mother and Musician follow Child's lead swishing the wall, Musician then attempts creation of *link in the loop*. She maintains accompaniment (*selective mirroring (modified))*, while Mother sings one long 'Swi-i-i-i-i-ish' (*activation contour*: *Stretched Up-swish in volume and pitch*) until Child reaches end of wall. Then a louder percussive chord is played, Mother sings 'Stop!' - creating *turbulen*ce (*relative acoustic startle*). Simultaneously, Mother pauses from following Child, lifts her hand from wall, palm open. Mother wriggles her fingers toward Child while vocalizing's various swishing sounds (*multimodal mirroring (vitality)*). Child pauses before continuing along another wall. The pattern repeats, with Mother following. Piano trills support her wriggling fingers as before. Child becomes excited, increasing walking pace, then becoming apparently aware that Mother and music follow him! Mother sings an animated responsive 'Swi-i-i-sh' as Child nears end of wall, culminating with another 'Stop!'

Link in the Loop

This pattern continues along another wall where Child reaches a metal cupboard. He bangs it; Mother and Musician immediately respond (with chords supporting Mother's imitation of Child's action) by *mirroring (vitality)* his banging. Suddenly, Child sits on floor; Musician plays glissando while singing 'falling down' *selective mirroring (initial).* Musician fills the space with an arpeggiated sequence while Mother waits for Child's next move, a *jazz gap* is created. Child stands up to bang cupboard again. Musician and Mother mirror his actions, while singing, 'Crash!'. There's a pause, Musician anticipates Child's return to bang cupboard, by playing chords and singing '1-2-3' and Mother joins in. Musician stretches anticipation on the '3' (*activation contour – Stretched Up-swish in volume and pitch*); adults sing '3'(rising intonation) before singing, 'Crash!' with lower voices aligned to Child's resumed banging.

Jazz Gaps & Activation Contours

Following '1-2-3.... Crash!', child looks directly at Mother. She's quick to sing '1-2-3..,' timing her 'Crash! to her Child's bangs on cupboard. Child's eyes fleetingly meet Mother's. Child bangs more fervently, Musician responds with lively rhythmic accompaniment while singing 'bam-ba-bam-ba-bam-ba'. Mother readily joins singing, edging closer, gently touching the Child's shoulders in time to the rhythm adding *multimodal mirroring (vitality).*

Orientation

Child returns to place his fingers on the wall leading to the cupboard; *rhythmic scaffolding* and *mirroring* resume. Musician accompanies with elongated 'swi-i-i-sh' over a crescendo as before. This time, as Child reaches half way point, Mother calls out '1... 2...' and, just before Child reaches cupboard, she shouts '3', creatively adding a *jazz gap*. Child gives Mother brief eye contact before re-launching into banging on cupboard; lively music ensues. Child stops banging, to return to the start of wall, so that the experience can be repeated. This time Mother gently presses Child's legs singing the 'bam-ba' rhythm (*layering the sense*s & extending her *mirroring (vitality)* from – and beyond - the high-energy "crash" and into calming tones and physical contact). There's a shared experience leading to the sense of a game, containing clear demarcated *contoured phrases*: O*rientation* is re-established each time when Child acknowledges Mother's presence; initially as Child makes sideways glances toward Mother, in the count "1-2-3", then as direct eye contact with Mother; Musician musically embodies Child's actions supporting Mother to assist in *developing synchrony*; There's a *jazz gap*, a crash (*peak*) and an *active withdrawal*, where Child and music diminish in vitality before reorientation. The variety of arousal experience (from the shared "Crash" down to the Mother's gentle physical contact and tones) enables a ranging *contour* – from exited, to calm, to active withdrawal, to reorientation and excited again. This describes the coregulatory nature of the narrative arc, throughout this, their shared journey.

Building a Game Together: Coregulatory Narrative Arcs

Over the course of therapy sessions, "1,2,3... Crash" becomes a recurrent strategy to engage Child. It later evolves into running from wall to wall and "crashing" into one. Child now makes direct eye contact with Mother before running. His face indicates his wish for her to follow him across the room, involving her in his experience. Mother catches him mid run, swings him around, they playfully descend to floor mattress. Child places his leg on Mother who, in response, teases him gently, "squashing" against his leg. Child laughs, then hurls his other leg at Mother who repeats the teasing on that leg. Child abruptly stands up returning to Crash routine.

Tonescapes: Extending Coregulation & Sensory Integration

Supplementary Figure 4. Case Example. Rhythmic Relating within Musical Interaction Therapy (MIT): Judit Elias-Masiques, Marie-Claire Howorth, & Dawn Wimpory.

MIT involves triadic working: Musician and familiar adult (e.g., parent) support the interactions of a child-client with their familiar adult (the person best placed to facilitate enhanced communication from that child - within and beyond therapy). Musician's role resembles live pianist's accompaniment in early cinemas.

Acclimatisation & Being Alongside

10 weeks in child-centered play therapy (CCPT). Client (4, boy, Autism (Therapeutic Needs)) + therapist. Playroom equipped with toys set forth in the CCPT treatment manual. Client has engaged in exploratory, simple object play for all sessions. Client is sitting in the sandbox, grabbing handfuls of sand, raises arms up and releases sand- a short, repetitious behavior *loop*.

Pre-Orientation

Therapist imitates client's physical actions, *selective mirroring (initial),* releasing fistfuls of sand alongside client. No observable reaction from client. The client's loop holds a behavior pulse, a roughly 4/4 rhythmic pattern of grab-rest-drop-grab-rest-drop. Therapist continues *mirroring* and adds *rhythmic synchrony scaffolding* (quiet, simple beat pattern):

Therapist:	"Meeee…" (Up-swish, vocal activation contour)	"MA." (Vocal beat)	"Loooo…" (Down-swoosh, vocal activation contour)	"LA." (Vocal beat)
Client:	Grabs sand	Drops sand	Grabs sand	Drops sand

Here the *activation contours* alternate between *Stretched Up-swish and Down-swoosh (in volume and pitch)* while the vocal beats are *accents*. No observable reaction from the client.

Orientating and/or When Things Feel Stuck...

Link in the Loop

Using a *follow-lead-follow* approach, therapist performs a playful experiment. Therapist attempts a *link in the loop*: just before the client releases the sand, therapist holds out hands to see if client will put sand into their hands. Client smiles and continues loop-grabbing handfuls of sand, raising arms up and releasing sand into therapist's hands.

Jazz Gaps & Activation Contours

Client and therapist engage in the sand play loop three times. Then, to encourage self-direction and an *orientation*, the therapist introduces a *jazz gap*: pausing the *rhythmic synchrony scaffolding* just after a vocal *activation contour*, "Meeee…" (to prime expectation of the on-beat moment - sand-into-hands), and instead, not putting hands out, waiting for a response.

Orientation

Client looks directly at therapist. Therapist responds to client by holding out hands and continuing their song (*rhythmic synchrony scaffolding)* and the *loop* continues.

Building a Game Together: Coregulatory Narrative Arcs

Therapist continues the *rhythmic scaffolding loop* and *jazz gap.* Therapist *modifies* their loop with *appropriate novelty* by grabbing fistfuls of sand and releasing sand. Client looks at therapist, smiles and laughs, and puts hands out for therapist to put sand in his hands. Therapist then begins *rhythmic scaffolding* by modifying their *rhythmic synchrony scaffolding (modified)* to with a higher pitch, higher volume 4/4 song: "I put sand...In your hands...Sand in my hand...Sand in your hand. A game develops along a series of *even phrases.* Each phrase following this pattern: and Tthe client and therapist engage in a *rhythmic loop* 1 (see box: *Preorientation* above) an initial well-defined loop (with non-verbal rhythmic scaffolding and a rhythmically-supported moment in which - then with a *jazz gap*, the therapist returns to the first *rhythmic scaffolding*, putsting hands out for client to put sand into hands);, Tthen, via a jazz gap, returning to the second *rhythmic loop* 2 (a more celebratory phrase, with more chaotic grabbing and release of sand, and verbal *rhythmic scaffolding*) after using a *jazz gap*. After 4 such phrases, the game reaches a natural *peak* of anticipation and excitement, and the client *pauses* for a few moments (*active withdrawal*). Client then *re-initiates* the game by grabbing fistfuls of sand. The sandplay game, *peak*, *pause*, and *re-initiation* are all moments of bidirectional synchrony and co-regulation.

Tonescapes: Extending Coregulation & Sensory Integration

Through several more months of engaging in CCPT, the client engages in variations of the phrase game: sandplay with cups, sandplay burying toys, sandplay with toys playing hide-and-seek.

Supplementary Figure 5. Case Example. Rhythmic Relating within Play Therapy: Karrie Swan.

Ten weekly sessions into music therapy. The room is equipped with musical instruments put aside on shelves and with good space on the floor. The client (6, boy, Autism (Therapeutic Needs)) stands restless beside the piano while the practitioner plays and sings a Hello song.

Acclimatisation & Being Alongside

For the first time, the client reaches out and plays the piano while looking around in the room with fleeting attention. The client plays with his right index finger, he plays fast without a steady pulse, and apparently without paying much attention to his own play. First the practitioner provides a slow *rhythmic grounding* with repeated chords from the Hello song (without playing the melody*)* but as the client does not react, the practitioner speeds up the tempo to match the client's tempo and restlessness, i.e. *multimodal mirroring (vitality)* combined with *rhythmic synchrony scaffolding (initial).* The client seems to enjoy the fast speed, his body moves a bit to the rhythm, he smiles and he gets more attentive towards his own playing looking at his index finger. After half a minute, the client's expression changes completely as he starts to play one key up at a time to the top of the piano and then back again. The smile disappears from his face, and he plays in a slower irregular pulse without the eagerness and vitality from before. It is as if he is stuck in the idea of playing one key up or down at a time in a loop, as opposed to the previous musical enjoyment.

Pre-Orientation

Orientating and/or When Things Feel Stuck...

In an attempt to make a *link in the loop*, while continuing to provide *rhythmic synchrony scaffolding (initial)*, the practitioner plays a bass keynote with the left hand as synchronously as possible with the client's play, alternating with a chord played with the right hand in between ('offbeat'). As well, the practitioner shifts between a few chords, so the harmony fits to the client's play, providing an *information-rich rhythm* that facilitates prediction and flow and acts as a '*repair*' of the client's unsteady pulse. The practitioner *follows the client's lead* but adds an element, the offbeat chord (*selective mirroring (modified)*), which provides an informational step-up, a *temporal cue*, which captures the client's attention and guides him closer to alignment in a shared basic rhythm.

Link in the Loop

The practitioner starts to accentuate the offbeat chord, and to play the chord right after the client's note. The chord and timing together give the play a marked forward-pushing quality, providing *rhythmic synchrony scaffolding (moderated) + an up-swish activation contour (using timing).* The practitioner continues to *play with turbulence and up-swish activation contours* as well as with big hand movements providing *multimodal cues*. Here, within the frame of *following-lead-follow*, the practitioner is quite *leading,* yet in a playful way!

Jazz Gaps & Activation Contours

Client looks at practitioner's hands and plays himself with slightly bigger hand movements in a steadier pulse. He smiles and seems to discover the connection between his own play and practitioner's. Next time client reaches the top of the piano, he breaks out of the fixed succession and starts to play fast and with joy, looking occasionally at practitioner's hands.

Orientation

In general, the client enjoys high tempo and intensity, but after a while, he shows signs of being restless and/or over-aroused. To balance the joy of high speed with built-in possibilities of regulating down without losing client's interest, practitioner makes a smooth transition from the fast 'off-beat' play mode to a slightly slower punctuated 'swinging' rhythm (a laid back quality), keeping the high volume and chord pattern, i.e. creating a *partly down-swoosh activation contour*. The first few times, client either hesitates or begins his play in fixed succession, unless the practitioner immediately switches back to the fast offbeat play. Over time, and after building an expectation of the shifts, client can stay a little longer in the swinging part before he wants the shift. It develops into a game – a *coregulatory narrative arc* – with *even phrases* starting with joyful and intense fast play with a lot of *rhythmic and harmonic turbulence*, followed by the more laid-back swinging part initiated by the practitioner, ending when the client initiates the next fast part. After some phrases, the game ends with a *peak* and laugh followed by a pause before the next *coregulatory narrative arc* begins.

Building a Game Together: Coregulatory Narrative Arcs

The game's two parts, the fast and the relatively slower one, remain. Gradually, however, the swinging part becomes more and more joyful as well. The client sits beside the practitioner at the piano and plays, hums/ sings or sways a bit from side to side to the swinging style of playing. Of course, the practitioner still uses many *activation contours* and *turbulence* to ensure variations and joyful surprises, but the calmer part provides moments of intersubjective intensity in a more intimate, subtle way than the fast off-beat part. The variation of rhythmic and intersubjective quality (fast and intense – calm and down-regulated) - initiated by the practitioner, yet quickly shared and developed by the client – offers a *tonescape* of emotional and arousal experience; a variety of *coregulatory narrative arcs* which enable the client with experiences of playful self-regulation (strengthening vagal tone, through practice; moving through excited and vulnerable, and back to calm, again and again).

Tonescapes: Extending Coregulation & Sensory Integration

Supplementary Figure 6. Case Example. Rhythmic Relating within Improvisational Music Therapy: Ulla Holck & Monika Geretsegger.

Sixth month of dance/movement therapy. Client (4, male, Autism (Therapeutic Needs)) + practitioner + mom (initially observing). Therapy room: includes mini trampoline, padded mats, recorded music. Client presents very limited verbal language, limited eye contact, sensory seeking in proprioceptive and vestibular sensations, demonstrates keen musical sense - fascination with musicals, classical music, and dance - particular interest in *Riverdance*. Mom has music and theatre background greatly supports client's interests.

Acclimatisation & Being Alongside

Client engages in beginning of session. We all follow ritual/*loop* he's established over course of six months: client enters, sits down, pulls shoes off, walks across the center of the room to the windows and back two times gazing around with soft focus as practitioner and mom stand patiently near the wall by the sound system. Using *rhythmic synchrony scaffolding (initial)*, practitioner claps out beat of client's walking step, in 4/4 meter, anchoring his actions. Client pauses, gazes towards practitioner and mom, and softly whispers "Riverdance," indicating he wants to dance to "Reel around the sun", the first song, Riverdance album. Practitioner puts it on. Client begins to dance to the looping melody. Using *rhythmic synchrony scaffolding (initial)* in 4/4, practitioner verbalizes his actions "Circle, circle, JUMP! JUMP!" adding louder accents on the *3 and 4* (i.e. on JUMP! JUMP!) and adding cross-modal *activation contours* (*Pulse in proximity,* as she shifts her *proximity* as walking towards him).

Pre-Orientation

Orientating and/or When Things Feel Stuck...

As the practitioner adds *rhythmic synchrony scaffolding (initial)*, to the client's looping dance, she becomes relevant to his *loop*, becomes part of his motivation.

Link in the Loop

Client momentarily pauses turning his body towards the practitioner as the music pauses, demonstrating his attunement to the melody. Practitioner pauses too, creating an exaggerated *jazz gap* (she leads into the expected response, leans into the *jazz gap,* with an *activation contour: Up-swish in proximity and embodiment* – looming in close for significance, bending her body towards him slightly and holding her body very still. Client, legs straddled, gently sways side-to-side almost imperceivably as a piano chord is loudly played and sustained in the music. Practitioner extends the *jazz gap* a moment longer to add emphasis.

Jazz Gaps & Activation Contours

In response to the rhythmically held *jazz gap*, the client rocks side-to-side with more accent, orienting his body more and closer towards practitioner. Developing her use of *activation contours*, (*now using Stretch in proximity and embodiment*) practitioner bends more deeply and rocks with client, softly stating "now we rock side-to-side" – *rhythmic synchrony scaffolding (developed)*.

Orientation

Turning his body closer towards practitioner, client initiates dancing dialogue by rocking with practitioner, with his arms bent at elbows. Practitioner develops *Stretch activation contours*, by fully embodying client's rocking motion - *mirroring (vitality)* - and takes his hands. They rock, and sway, following undulating contours of the musical melody, together co-creating an *even phrase* in *simultaneity*. As dance progresses their bodies sway and bend more deeply, adding balancing on one foot while gliding foot on the floor. The balancing actions are in proximity, solidify their connection as they support each other's balancing poses, shifting weight forward, side, and backward when extending moving leg to the back, side, or front of their bodies. Client maintains eye contact through peripheral vision. When the melody of the song changes again to faster rhythmic pulse, client immediately changes dance moves, dropping the handholding, galloping around whole room with more vigour. Practitioner develops *mirroring (vitality)* to follow in response, at times verbally narrating their actions and adding *pendulous phrasing* qualities to their actions, creating *exaggerated accents (vocalizations accented in pitch, volume, and timbre)* that follow their dance moves in rhythmic synchrony to the music.

Building a Game Together: Coregulatory Narrative Arcs

Music becomes softer, more melodic. Mom creates a *trio* as she joins the dance (entering with an *activation contour: Stretched Up-swish in physical proximity - looming in playfully)*. Client orients to Mom. Practitioner steps back, supporting their duet. Using *layering the senses*, Mom lifts client into her arms. Client wraps an arm around Mom's back and legs around her waist, creating more proprioceptive sensation. Holding hands, they extend and glide their arms together. Client orients his vision at times out toward the practitioner, tracks their arm dance and provides fleeting eye contact with Mom, while smiling. These dances with Mom and practitioner build for next year, as client brings in more songs from musicals each session.

Tonescapes: Extending Coregulation & Sensory Integration

Supplementary Figure 7. Case Example. Rhythmic Relating within Dance Movement Therapy: Suzi Tortora.

Triadic session: Child-Client (4 years boy, Autism [Therapeutic Needs]) + Mother (experienced in MIT) + Musician (Musical Interaction Therapist). Low lit quiet room *without* toys. Mother follows Child who plunges himself onto sofa while Musician plays ascending sixths to provide *selective mirroring (initial) – cross-modal mirroring (vitality)*. Child twists and turns, finally settling almost upside down, Mother bounces herself down beside him. Child appears to enjoy the sensory feedback of their combined movement on sofa.

Acclimatisation & Being Alongside

Child is bottom-bouncing on sofa, his bouncing propels his outstretched arms through the air and he shouts excited vocalizations. Musician latches onto his rhythm now regular enough for *rhythmic synchrony scaffolding (initial)*. Mother naturally joins Child in bouncing, whilst singing, 'bouncing, bouncing, bouncing, bouncing, bouncing on the sofa!' until Child stops. Musician interjects with sustained chord (*appropriate novelty*); Child looks up and around room, rather than at anyone. He clenches his hands open and shut a few times, whilst vocalizing. Mother repeats his vocalizations, positioning herself half facing him, opening and shutting her hands in responsive *selective mirroring (initial) & cross-modal mirroring (vitality).* After a pause, Child vocalizes again, his serious tone and concentrated facial expression provoke Mother to answer similarly. Musician uses slow sustained chords capturing their changed pace; *rhythmic synchrony grounding*. This sustained soundscape supports antiphonal vocalizations between Child and Mother. Mother crouches in front of child to face him, level with his eyes but not too close. As the rhythm becomes more predictable, *rhythmic synchrony scaffolding (initial)* is re-established and elements of their loose conversation are intertwined within the musical accompaniment's pitch contours (*rhythmic synchrony scaffolding (modified)).* Child returns to his initial mode of energetic bouncing on sofa, Mother seizes the opportunity to re-join him. Lively music and *rhythmic synchrony scaffolding (initial)* are reintroduced.

Pre-Orientation

Orientating and/or When Things Feel Stuck...

Link in the Loop

Musician and Mother continue singing about Child's actions (with Mother markedly 'bouncing' and singing that word – *multimodal mirroring (vitality)*). This is repeated until they introduce a *jazz gap* (*pause*), where Mother replaces word 'sofa' with a gasp; she skips a bounce. Child looks toward Mother, though without eye contact. She sings to him, quietly building to a crescendo (*activation contour, Stretched up-swish in volume and pitch*) before repeating *the jazz gap*. Child squeals excitedly and pattern is repeated. Musician accompanies this, then adds novelty by increasing tempo. This encourages Mother to sing faster, leading to another *jazz gap*.

Jazz Gaps & Activation Contours

Pausing in the *jazz gap* gives rise to a peak of experience, before withdrawal and restarting. On the final occasion, Child vocalizes in the *jazz gap*, stops and looks around the room then Mother glides her voice to make similar noises in response. Child then runs toward, but stops short of touching, keyboard. Mother has followed and positioned herself, crouching at Child's height, between keyboard and Child, who then faces her to give momentary direct eye contact. Child laughs. Mother laughs and once again his eyes catch hers. Child lets out cheeky giggling with excited gleeful squeals.

Orientation

Musician responds to their eye contact, with a staccato chord, creating an *acoustic startle response*. The moment is charged with *turbulence*, Mother jumps up and Child quickly runs back to sofa. Mother follows playfully, while singing: 'run run run'. As Child returns to sofa, Musician plays light joyful music to highlight the playfulness of their interaction. The bouncing is repeated, along with the song and *jazz gap*. Then, Child appears to feel safe to run towards the keyboard once again while (though he knows, from experience that the music and singing become quiet if he touches its keys). Mother excitedly follows him; eye contact occurs again. Child smiles and laughs whilst running back to sofa to crash into it with a squeal, followed by Mother who copies the squeal. There are many follow-on runs back to sofa that develop into a chase (a game defined by a series of *even phrases*). Mother enhances the chase with outstretched hands, poised ready to tickle him on sofa. She tickles and when the tickling reaches its peak, there's a natural lull. Mother expels long sighs, and, in the calm, she sings 'Go to sleep, rest your eyes' while stroking his head. Musician supports the melody and mood.

Building a Game Together: Coregulatory Narrative Arcs

The "bouncing on the sofa" game enables *coregulatory* experience of *narrative arcs* – taking Child and Mother through arousal and down to calm in cycles shared in synchrony. The game becomes a cornerstone for various interactive routines. Mother's replacing of "sofa" with a gasp, then gradually introducing words like 'floor' (incorporating relevant actions), creates new *links in the loop*. There's increased eye contact to Mother, as Child becomes familiar with the *jazz gap game*. Subsequent MIT sessions include development of the game whereby a tickle-chase-pattern emerges when Child runs back toward sofa 'safety'. Child runs around sofa with Mother in pursuit, singing, 'I'm gonna get you!'. Musician accompanies Mother's singing to Child. When Child returns to sofa, he buries down into a cushion and Mother uses a slower, sing-song, teasing voice: 'I can s-e-e you'.

Tonescapes: Extending Coregulation & Sensory Integration

Supplementary Figure 8. Case Example. Rhythmic Relating within Musical Interaction Therapy (MIT): Judit Elias-Masiques, Marie-Claire Howorth, & Dawn Wimpory.

MIT involves triadic working: Musician and familiar adult (e.g., parent) support the interactions of a child-client with their familiar adult (the person best placed to facilitate enhanced communication from that child - within and beyond therapy). Musician's role resembles live pianist's accompaniment in early cinemas.

Early in Therapy. Play Therapy room: all equipment on covered shelves, lamp light (fluorescent overhead off), "do not disturb" sign on, as minimal as possible. Client (5, boy, Autism (Therapeutic Needs)) + practitioner. Start: client sitting on cushion and vocalising (irregular), practitioner sitting not-too-close, open posture, eye-gaze near, not direct eye-contact. Client has been exploring different places to sit and vocalising for 3 previous sessions. Practitioner has been alongside, sometimes *mirroring* the vocalisation with light singing.

Acclimatisation & Being Alongside

Client is vocalizing (irregular) + left hand, all fingers press and slap against palm in a clench-release (slow regular rhythm). Practitioner adds vocal *rhythmic synchrony scaffolding* (iambic pentameter "te-tisss, te-tisss, te-tisss, te-tisss"; "*tisss*" reflects the duration of the client's fingers clench-and-release) (very quite starting volume level). Continues for 10 mins. Practitioner slowly moves close to client, staying floor level. Client has bare feet (practitioner, previous knowledge = client likes firm physical contact). Adds *multimodality* to *scaffolding* (on "te" = gentle contact, both hands on client's feet, on "tisss" = *activation contour*, stretch in intensity (physical contact), i.e, contact gets steadily firmer in a contour culminating in the client relaxing his hand. This *activation contour* in physical contact uses *mirroring (vitality)* – mirroring the contoured vitality of the client's hand clench-and-release. This loop continues For 5 mins.

Pre-Orientation

Orientating and/or When Things Feel Stuck...

On ever fourth "te-tisss" in the iambic pentameter, practitioner Replaces the "tisss" with a louder, higher-pitch "PING" vocalization (*relative acoustic startle*, relative to the quite starting volume level) and, on this beat only, practitioner waggles the client's feet a little. The "PING"/feet waggle is a *turbulent* beat now included in the pulse – with the aim of becoming a link in the loop. Continues for a few mins.

The loop now looks as follows:

	C.Hand Clench/ Release		C.Hand Clench/ Release		C.Hand Clench/ Release		C.Hand Clench/ Release
te-	tiss	te-	tiss	te-	tiss	te-	PING
Foot contact	*Foot contour*	Foot contact	*Foot contour*	Foot contact	*Foot contour*	Foot contact	*Foot waggle*

Link in the Loop

After a few mins, practitioner uses a *jazz gap*:

	C.Hand Clench/ Release		C.Hand Clench/ Release		C.Hand Clench/ Release		C.Hand Clench/ Release
te-	tiss	te-	tiss	te-	tiss	te-	
Foot contact	*Foot contour*	Foot contact	*Foot contour*	Foot contact	*Foot contour*	Foot contact	**JAZZ GAP**

Jazz Gaps & Activation Contours

Client initially ignores the *jazz gap* for a few iterations of the loop. Then, in response to a *jazz gap*, he looks up at the practitioner and pokes them in the head (gently)!

Orientation

Practitioner smiles & laughs (very congruently!) with surprise. Client stops his vocalization + hand clench/release. Practitioner immediately continues the *rhythmic scaffolding* loop, using the *jazz gap* to wait for a response – which happens immediately, another poke in the head! Practitioner *modifies* their loop with *appropriate novelty* – they slowly edge away from the client, sliding backwards across the floor on their bottom. Client smiles and follows, needing to catch up with the practitioner to complete the poke-in-the-head at the *jazz gap* moment. Practitioner tries *modification* - gently pokes the client on the forehead to match the client's action – this does not work and upsets the client. Sensitively using *follow-lead-follow*, the practitioner does a theatrical shrug, says "silly me", and immediately returns to the loop. The game develops along the lines of a series of *even phrases*, in which client and practitioner run together (client chasing practitioner) for the duration of the rhythmic loop – then with the *jazz gap*, the practitioner turns around theatrically, client smiles and pokes practitioner! 3 or 4 phrases and the game reaches a natural *peak*. Client and practitioner rest a moment. Then client *reinitiates* by starting to chase. The chase, turn-around-poke-moment, *peak*, *active withdrawal*, and *re-initiation* are all moments of bidirectional synchrony and fun.

Building a Game Together: Coregulatory Narrative Arcs

Over the course of a few months of play therapy sessions, this initial moment of contact and *even phrase* game structure develops to include variations: soft ball instead of head-poke; soft-ball turn-taking in a *pendulous phrase* structure; practitioner running, then hiding in the tent; practitioner and client in the tent (mood change, practitioner introduces quiet *grounding rhythm* with whisper-beats and "tiings", this leads to very gentle synchrony, playing with "spider fingers").

Tonescapes: Extending Coregulation & Sensory Integration

Supplementary Figure 9. Case Example. Rhythmic Relating within Play Therapy: Stuart Daniel.

Early in music therapy. Room equipped with musical instruments on shelves, with good space on the floor. The client (4, girl, Autism (Therapeutic Needs)) walks around slowly, sometimes pausing to touch an object's surface or the wall, and occasionally vocalises, uttering fragile, screeching sounds. Practitioner sits on a chair, gently plucking the strings of a guitar in a basic 4/4 meter (alternating between A minor and E major) to provide a calm atmosphere (*rhythmic grounding*).

Acclimatisation & Being Alongside

As the client continues moving around and vocalising, the practitioner keeps playing, picking up small features of the client's behaviour such as her stroking a surface, and incorporating these through *accents* in the otherwise steady pulse of the guitar-playing – evolving from the grounding into *rhythmic synchrony scaffolding (initial)* and using *selective mirroring (initial)*. The client sometimes pauses her movements for brief periods of time, possibly listening to the practitioner's sounds, but otherwise shows no apparent sign of orientation towards the practitioner or the playing.

Pre-Orientation

Orientating and/or When Things Feel Stuck...

Link in the Loop

After a while, the client's movements and the music provided by the practitioner seem to mutually complement each other. In an exploratory attempt to understand and reflect the quality of the client's emotional state (frightened? agitated? discomfort? insecurity?), the practitioner uses *mirroring (vitality)* within a series of different *activation contours*: starts with vocal imitation of the client's screeching sounds, but using a soft instead of a screechy voice, and adding small, soft melodic *activation contours*: a slow downward contour for one utterance (*stretched down-swoosh in volume, pitch, and timbre*), and an upward, question-like contour in the next (*stretched up-swish, again in volume, pitch and timbre*), with long pauses (and ongoing guitar-playing) in between – *selective mirroring (modified)*.

Jazz Gaps & Activation Contours

As the practitioner utters a slightly stronger vocal response to one of the client's vocalisations, the client looks at the practitioner, briefly meeting their gaze. The client approaches the practitioner and cautiously touches the guitar strings, almost without eliciting a sound from them. The practitioner accompanies this with a few gentle contoured vocalisations, like with a sense of wonder.

Orientation

After some sessions with a similar succession of events, the client starts moving her hand across the guitar strings, now with sound. Practitioner keeps using the left hand for maintaining the harmony of the chords the client is now stroking in a regular pulse. Both client and practitioner start moving their bodies, swaying a little from side to side. As the client stops playing, practitioner takes over to keep the regular forward-moving pulse of the movement in the musical accompaniment – *rhythmic synchrony scaffolding (modified)*. After a while, practitioner adds the vocal *activation contours* again, but now in a more rhythmic, song-like manner, following the pulse of the guitar playing. The singing matches the energy and intensity of the client's movements and expression – *selective mirroring (vitality)*. As the short melody comes to an end, practitioner pauses the playing, providing space for the client to respond. After a short phase of self-regulatory *withdrawal*, the client looks at the practitioner and starts stroking the strings again (a clear *re-orientation*). Using a sensitive approach of *follow-lead-follow*, both continue to build this game of *coregulatory narrative arcs* with variations in volume/tempo/intensity, in synchronous simultaneity and complementarity.

Building a Game Together: Coregulatory Narrative Arcs

Over the next sessions, and as the client's capacity for attention and joint engagement expands, the vocalisations develop into a little song with practitioner adding lyrics, using both interaction partners' names and simple descriptions of what they are doing – always matched in vitality to the client's current expression, and often initiated by client through deliberate and inviting use of her previously screeching, now much softer vocalisations. The song and its rhythmic melody serve as a safe, predictable base of departure for experimenting with variations, corresponding bodily movements to facilitate sensorimotor integration (*multimodality mirroring (vitality)*), and playful violations of expectations using exaggerations, sudden changes, "wrong" chords or words, or *jazz gaps*. *gaps*. Our *co-vocalising*, and *playful teasing*, has developed into a highly interactive game based around turn-taking in *initiation, orientation, and development of shared rhythm* (across a series of *pendulous phrases*).

Tonescapes: Extending Coregulation & Sensory Integration

Supplementary Figure 10. Case Example. Rhythmic Relating within Improvisational Music Therapy: Monika Geretsegger & Ulla Holck.

Fourth month of Dance Movement Therapy. Client (3-year-old male, Autism (Therapeutic Needs)), sparce verbalizations, very musical, limited eye contact, tactile defensiveness. Large therapy room - props on/against walls: coloured silk and nylon sheer scarves & streamers, pillows; on shelving - instruments, stereo; large open floor space. Client and parent enter. Parent sits near entrance; client immediately runs across room, throws self on floor holding CD music booklet; practitioner follows client and pauses, standing near client.

Acclimatisation & Being Alongside

CD music plays. Practitioner watches client's nonverbal cues: lying on back, knees opening and closing, tensely, clutching CD book. After a few minutes, practitioner crouches down to eye-level, calmly stating, "time to have book back… time for dancing". Client immediately protests, flinging legs, rolling on back, steps to stand, running towards practitioner, flinging body down to ground prone, kicking/screaming. Using *Rhythmic Synchrony Scaffolding (initial)*, practitioner tries matching rhythm of client's kicks, rhythmically stamping her feet near his torso. Next, practitioner uses *Selective Mirroring (initial*), falling to floor in same manner of client but then lying on side, pausing quietly. Client pauses kicking, pushing ground with forearms, lifting body up, gazing toward practitioner. Practitioner mirrors exactly, both sit up and pause gazing at each other. Client exclaims "Boouuk back". Practitioner says gently, "no more book..." Client rolls to hands and knees then on to back. Practitioner crawls closer, sits up, legs folded under her, near client. Here practitioner is not copying client's actions, but instead *Mirroring (Vitality)* to reflect an essence – the physical/energetic size and shape of client's communication. Introducing *Rhythmic Grounding*, practitioner taps beat of CD music, on her lap. Client sweeps his legs onto practitioner's lap. *Follow -lead – follow*: practitioner taps client's feet on the floor to the beat bringing multi-modality (*Layering the Senses*) into the *Rhythmic Grounding*.

Pre-Orientation

Orientating and/or When Things Feel Stuck...

Client initially follows, matching the CD beat and practitioner's *Rhythmic Grounding* through supportive leg actions. There's a pause in this interaction as client twists his body, rolling onto his back – his legs bent. Client begins vocalizing more quietly and practitioner again taps client's feet on the floor – but this time uses *Rhythmic Synchrony Scaffolding (initial)* to match client's vocalizations. Practitioner now rises on her knees, arching her body over client's torso to have more direct eye contact and continues Rhythmic Scaffolding. Within the scaffolding, practitioner adds the phrase, "time for dancing…" Client makes eye contact. Within the multimodal scaffolding – matching the client's vocalizations – the practitioner's words have become a *Link in the Loop*; important to the completion of the shared rhythm.

Link in the Loop

And...

After a few iterations of the shared rhythm, practitioner pauses after "time for dancing…" – introducing a *Jazz Gap*.

Jazz Gaps & Activation Contours

Client rolls onto side and both client and practitioner extend the pause. Client then stands and runs across the room in a prancing rhythm.

Orientation

Client prances to center of the room. Tempo of music softens, and client slightly calms - apparent in softer vocalizations and releasing tension in legs. Practitioner takes client's legs again (with knees facing ceiling), this time slowly rocking them side-to-side (*Rhythmic Synchrony Scaffolding (extended)* - attuning to slower music and client's mood). Next, client looks towards Mom, stating "change, change" and begins more actively kicking and extending his legs. Developing the scaffolding to match, practitioner pulses client's legs, alternating left and right legs in a 2/4 marching accented rhythm, stating "change, change, change..." Client states "CHANGE, CHANGE…" as he modifies practitioner's actions by extending his legs together into her lap in an extension – flexion rhythmic pattern creating deep sensory input. Client calms, practitioner lifts client and places him in Mom's lap. Client sits for a moment, then flings his body arching backward over Mom's arm and vocalizing strongly. Extending and exaggerating the motion (and *Layering the Senses*), practitioner lifts him, holding him upside down creating increased vestibular and proprioceptive sensorial stimulation, then places client in Moms arms. Mom shapes her whole body, legs and arms around client rocking him side-to-side as client shapes his body into Mom. After a while in the containment of Mom's rocking, practitioner suggests, "Can we dance?" Client says, "okay". Practitioner changes music to stronger 4/4 rhythmic beat. Mom stands, begins side-step skipping dance. Client taps foot to beat, practitioner stands near, tapping drum and her foot to his beat (*Rhythmic Synchrony Scaffolding (extended)*). Mom and practitioner follow client's rhythmic dance-moves around room. The client has moved through calm, to slightly dysregulated and vulnerable, through to social engagement and a truly shared, playful dance with both adults. This *co-regulatory narrative arc* was held through sensitive movement, rhythm, and attachment play.

Building a Game Together: Coregulatory Narrative Arcs

Tonescapes: Extending Coregulation & Sensory Integration

Supplementary Figure 11. Case Example. Rhythmic Relating within Dance Movement Therapy: Suzi Tortora.

Triadic session: Child-Client (5 years, girl, Autism (Therapeutic Needs)) + Mother (experienced in MIT) + Musician (Musical Interaction Therapist). Naturally lit room *without* toys, contains: foam floor mats, cupboard mattress, sofa and cushions. Child enters on barefoot tip-toes, immediately interested in floor mats. Mother is attentive to the delight Child is experiencing through barefoot contact with mats. Mother takes off own shoes to join Child. Musician offers ragtime accompaniment.

Acclimatisation & Being Alongside

Child develops a *closed loop* characterized by walking on tiptoes over soft, ribbed floor mats. She walks from one side of room to opposite while vocalizing and flapping arms. Mother walks alongside Child. There is no discernible rhythm in Child's actions but ragtime accompaniment in place provides a 4/4 *rhythmic grounding*. Mother quietly hums low notes as *selective mirroring (initial) of Child's vocalizations*. Child ignores both Musician and Mother. Musician waits, giving Child more time to adapt without her becoming overwhelmed, as Mother continues to support her. After a while, Child sits cross-legged on floor, rocking back and forth while vocalizing and flapping hands. Mother sits diagonally next to her, half facing Child, also rocking back and forth using *selective mirroring*.

Mother's Voice	Hummmm Up-swish *activation contour*	Hummmm Down-swoosh *activation contour*	Hummmm Up-swish *activation contour*	Hummmm *Down-swoosh activation contour*
Piano (4/4)	Upper note pitch	Lower note pitch	Upper note pitch	Adjacent lower note pitch
Mother's Actions	Mother rocks back and forward in time to the humming and piano			

Pre-Orientation

Orientating and/or When Things Feel Stuck...

Mother introduces a *Link in the Loop,* by exaggeratedly flopping down, extending hands, and touching her forehead to the soft floor on her fourth rock. Musician matches Mother's vocal *up swish* intonation as she sings a third "hummmm". Musician responds to this new *link*, creating a crescendo and decelerating process for Mother to replace her final "hummmm" (the up-swish extended hummm, the exaggerated flop, and crescendo all acting as a step-up guide towards the onbeat...) with a "Splat!"and an accentuating chord.

Mother's Voice	Hummmm (Up-swish)	Hummmm (Down-swoosh)	Hummmm... (Up-swish)	Splat!!!
Piano (4/4)	Upper note pitch	Lower note pitch	Upper note pitch. Crescendo & rising scale stretching the 3/4 pulse, slowing down	Chord
Mother's Actions	Mother rocks back and forward			Mother flops to floor

Link in the Loop

Musician cues Mother to introduce a *jazz gap*, by replacing "Splat!" with silence and by Mother *not* flopping down. Child looks directly at Mother during this *jazz gap* before rolling on the mat. Mother *then* flops down rolling parallel to Child. Musician employs cross-modal *selective mirroring (initial)*, emphasizing Mother's actions. Child responds to Mother's flopping and rolling with a high-pitched excited vocalization and hand-flapping, that Mother copies. Musician imitates by *mirroring the vitality* of interaction (accelerating dense rhythms, broad in pitch, high level *turbulence*), before playing sustained vibrant chords. The music provides space for Mother and Child to explore intimacy. Mother massages Child's back. There's a brief feeling of meaningful shared attention and meaning; a moment resonating excitement. Child touches her own feet, Mother lies down alongside her and touches Child's feet with her own bare toes...

Jazz Gaps & Activation Contours

Child sits up returning to initial rocking pattern. But now, when Mother withholds the flopping down action for a few beats (*jazz gap*), it is noticeable that Child makes eye contact with her on several occasions. Child smiles indicating that she is clearly enjoying the interaction, although her smiles and eye contact are not yet simultaneous.

Orientation

During one of the pauses, Child foregoes eye contact and rocks forward until her forehead touches floormat, flopping similarly to the Mother's action. Musician responds with a descending glissando to a low chord and laughs, along with Mother. As Mother flops down, Musician creates a melodic and rhythmic motif, allowing the moment to be fully embraced. Then Musician returns to 4/4 pattern, encouraging opportunities for the sequence to be repeated several times. The humming becomes integral to the lulling "sing-song" motion created in the rocking. Anticipation builds towards the 'Splat!', *mirroring the vitality* expressed by Child's vocalizations and hand flapping. This *contoured phrase* is characterized in the following manner:

	Orientation	Developing Synchrony			Jazz Gap	Peak
Mother's Voice		Hummm (Up-swish)	Hummm (Down-swoosh)	Hummmm... (Up-swish)	Vocal gasp	Splat!
Piano (4/4)		Upper note pitch	Lower note pitch	Ascending pitch. Ascending textured chordal scalic pattern over pedal bass	Dominant 7th Chord	Chord resolve
Dyad's Actions	Child & Mother realign for the phrase	Mother rocks back and forward			Child: brief eye contact, or initiates flop	Mother & child flop to floor

Building a Game Together: Coregulatory Narrative Arcs

Over subsequent sessions, this routine becomes one of Child's favorites. Musician slowly introduces changes through *rhythmic synchrony scaffolding (extended)*, Mother adds gentle touch as part of game, *layering the senses* (i.e., forehead to forehead touching after flopping to floor, extending modality for the *rhythmic synchrony scaffolding*). New *links in the loop* are explored: instead of flopping, Child and Mother roll lying sideways on floor. Later, Mother blows raspberries on Child's skin in between rollings, and the dyad ends up rolling together, with Child laughing whilst being snugly held. Both gain a sense of shared experience.

Tonescapes: Extending Coregulation & Sensory Integration

Supplementary Figure 12. Case Example. Rhythmic Relating within Musical Interaction Therapy (MIT): Judit Elias-Masiques, Marie-Claire Howorth, & Dawn Wimpory.
MIT involves triadic working: Musician and familiar adult (e.g., parent) support the interactions of a child-client with their familiar adult (the person best placed to facilitate enhanced communication from that child - within and beyond therapy). Musician's role resembles live pianist's accompaniment in early cinemas.

20 weeks in child-centered play therapy (CCPT). Client (4, boy, Autism (Therapeutic Needs)) + therapist. Playroom equipped with toys set forth in the CCPT treatment manual. Client and therapist have a strong therapeutic relationship. Client enjoys *literal-association object-play* with toy soldiers, doll house, and doctor kit.

Acclimatisation & Being Alongside

Client puts 12 green army soldiers in a line, touches each soldier's gun and then knocks them over, making shooting noises-engaging in a *loop of literal-association object-play*, a repetitious behavior loop. The client's looping is largely arhythmic (yet each loop is completed in a similar short duration).As such, the therapist uses *rhythmic grounding* with a 4/4 "we are soldiers, bum bad um bum bum bum bum" – ending her rhythmic loop at the moment the client finishes his, as he touches the last soldier's gun. The client pauses, then looks at self in the mirror in front of himself and smiles. Client appears happy about the therapist's *rhythmic grounding*.

Pre-Orientation

Orientating and/or When Things Feel Stuck...

Link in the Loop

Client engages in his behavioral loop while appearing to enjoy the integration of therapist's use of *rhythmic grounding.* To encourage self-direction and an *orientation*, the therapist introduces a *jazz gap,* pausing the *rhythmic grounding* once client has touched the last soldier.

Jazz Gaps & Activation Contours

The *jazz gap* appears to influence the client as client looks at the therapist and states: "again." The therapist responds to this *orientation* by immediately re-starting the *rhythmic grounding*.

Orientation

Each *loop* of placing soldiers in a line, touching each soldier's gun, and knocking the soldiers down represents an *even phrase* shared by the client and therapist in *simultaneity*, creating a shared experience. The development of the next *phrase* is led by the client. Over 3 iterations, client says: "more" and therapist responds with *rhythmic synchrony scaffolding (extended)* - louder vocal *accents* and more *turbulent* sound effects. The *coregulatory narrative arc* of the game reaches peak when the client excitedly grabs all the soldiers and tosses them on the table. Client *withdraws* for a few moments (in active self-regulation) and then *re-orientates* by placing soldiers in a line, touching each soldier's gun, and then looking at therapist, stating "more." Therapist responds by using *rhythmic grounding* - this time adding a 4/4 accompaniment to their vocalizations by drumming on the floor with their hands.

Building a Game Together: Coregulatory Narrative Arcs

Through several more months of engaging in CCPT, the client engages in self-directed play: using the doctor kit with the therapist, playing with pretend food, and giving this therapist pretend food/drink. Each play experience offers *co-regulatory narrative arcs* with variations in arousal and mood, forming a *varied tonescape.*

Tonescapes: Extending Coregulation & Sensory Integration

Supplementary Figure 13. Case Example. Rhythmic Relating within Play Therapy: Karrie Swan.
